# Revision of the southern Andean genus *Sadocus* Sørensen, 1886 (Opiliones, Gonyleptidae, Pachylinae)

**DOI:** 10.3897/zookeys.1025.57806

**Published:** 2021-03-22

**Authors:** Marília Pessoa-Silva, Marcos Ryotaro Hara, Ricardo Pinto-da-Rocha

**Affiliations:** 1 Departamento de Zoologia, Instituto de Biociências, Universidade de São Paulo, São Paulo, São Paulo, Brazil Universidade de São Paulo São Paulo Brazil; 2 Escola de Artes, Ciências e Humanidades, Universidade de São Paulo, Av. Arlindo Béttio, 1000, Ermelino Matarazzo, 03828-000, São Paulo, SP, Brazil Universidade de São Paulo São Paulo Brazil

**Keywords:** Argentina, Chile, harvestmen

## Abstract

Species of the genus *Sadocus* Sørensen, 1886 are conspicuous gonyleptids that occur in Chile and Argentina. Here, the genus is revised for the first time and the cladistic analysis based on morphological characters does not corroborate its monophyly unless a phylogenetically unrelated species is excluded (explained further on). A new classification is proposed for the seven species left in the genus and considered valid, of the 13 nominal species previously recognized. Two out of the seven valid species are considered as species inquirendae: *Sadocus allermayeri* (Mello-Leitão, 1945) [= *Carampangue allermayeri* Mello-Leitão, 1945] and *Sadocus nigronotatus* (Mello-Leitão, 1943) [= *Carampangue nigronotatum* Mello-Leitão, 1943]. The following synonymies are proposed: *Sadocus bicornis* (Gervais, 1849) [original combination = *Gonyleptes bicornis* Gervais, 1849] is a junior synonym of *Sadocus asperatus* (Gervais, 1847) [= *Gonyleptes asperatus* Gervais, 1847]; *Sadocus conspicillatus* Roewer, 1913, *Sadocus exceptionalis* (Mello-Leitão, 1946) [= *Araucanoleptes exceptionalis* Mello-Leitão, 1946] and *Sadocus guttatus* Sørensen, 1902 are junior synonyms of the valid name *Sadocus polyacanthus* (Gervais, 1847) [= *Gonyleptes polyacanthus* Gervais, 1847]; and *Sadocus calcar* (Roewer, 1913) [= *Lycomedes calcar* Roewer, 1913] is a junior synonym of the valid name *Gonyleptes horridus* Kirby, 1819. *Sadocus brasiliensis* Soares & Soares, 1949 is not congeneric with Argentinean/Chilean species of the genus according to the cladistic analysis and is here synonymized with *Discocyrtus catharinensis* (Mello-Leitão, 1923 [= *Sadocus catharinensis* Mello-Leitão, 1923]).

## Introduction

Harvestman systematics has advanced greatly in the last few decades, especially in the Neotropical region, with many supraspecific groups being recently revised, such as for example Stygnidae Simon, 1879 (Pinto-da-Rocha 1997), Sodreaninae Soares & Soares, 1985 (Pinto-da-Rocha and Bragagnolo 2010), Goniosomatinae Mello-Leitão, 1935 (DaSilva and Gnaspini 2010), Hernandariinae Sørensen, 1884 (DaSilva and Pinto-da-Rocha 2010), among others. Gonyleptidae Sundevall, 1833, the largest Neotropical family in number of species, includes two taxonomically challenging and species-rich subfamilies pending revision: Gonyleptinae Sundevall, 1833 and Pachylinae Sørensen, 1884. The lack of revisions is possibly due to the considerable number of species and their great morphological variation.

Pachylinae is the most species-rich subfamily of Gonyleptidae, and is currently considered polyphyletic (Pinto-da-Rocha 2002; Pinto-da-Rocha et al. 2014; Benavides et al. 2021). A phylogenetic analysis, based on molecular data (Pinto-da-Rocha et al. 2014), recovered a clade including *Pachylus*Koch (1839), the type genus of the subfamily. This clade was named Pachylinae sensu stricto and includes mainly Chilean species. This result was the first step towards the dismemberment of this large subfamily into smaller monophyletic units.

The sister group of Pachylinae sensu stricto is a clade that includes the genus *Sadocus* Sørensen, 1886, composed of rather large-sized (5.5–13.8 mm of dorsal scutum length) and colorful harvestmen. Although conspicuous and relatively common in Chilean preserved areas, it was never revised in more than 130 years of existence. Historically (see below), the genus has been subjected to many taxonomic acts, resulting in confusing species identities. One has to use poor (for modern standards), hundred-year-old descriptions to identify a given species. In addition, similar species are difficult to distinguish, raising doubts about their identities. Therefore, the revision of *Sadocus* focuses on determining the identity of the included species, which in turn will allow further understanding of their relationships, and more precise inferences of their distribution and diversity (Acosta 2002). The goals of this article are also to test the monophyly of the genus and propose a classification based on cladistic analysis.

### Historical aspects of *Sadocus* Sørensen, 1886

The history of *Sadocus* Sørensen, 1886 can be quite confusing because many of its species were described before the proposition of the genus. Therefore, this historical section mentions many species in different genera and subfamilies that were later transferred to *Sadocus* (Kury 2003; Kury et al. 2020a, b), as explained further on in this article.

Guérin-Méneville (1844) described the eldest species related to *Sadocus*, *Gonyleptes planiceps* in Cuvier’s ‘Iconographie du Règne Animal’. However, the publication of this issue was delayed, and Gervais’ (1842) “redescription” was actually published first. Gervais (1842) did credit the authorship of *G. planiceps* to Guérin-Méneville, and he redescribed that species in 1844. Gervais also described the next five species relevant to *Sadocus*: *Gonyleptus asperatus*, *G. polyacanthoides*, and *G. polyacanthus* in 1847, and *G. bicornis* and *G. subsimilis* in 1849. Butler (1873) mistakenly proposed *G. subsimilis* as a senior synonym of *G. polyacanthoides*. A year later, Butler (1874) described *Gonyleptes funestus*. In 1884, Simon transferred *G. planiceps* to *Pachylus*.

In 1886, Sørensen proposed the monotypic genus *Sadocus*, to include the type and new species *S. vitellinosulcatus*. In 1899, Loman described *Discocyrtus calcitrosus* and *Gonyleptes platei*, which are relevant to *Sadocus*, and transferred *G. funestus* to *Discocyrtus* Holmberg, 1878.

In 1902, Sørensen: (i) synonymized *S. vitellinosulcatus* and *G. platei* Loman, 1899 with *G. polyacanthus*; (ii) created the new genus *Lycomedes* (without indication of a type species), to which he transferred *G. asperatus*, *G. bicornis*, *D. calcitrosus*, *D. funestus* and *Pachylus planiceps*; and (iii) described *Sadocus guttatus*. On that paper, Sørensen placed these species in Gonyleptidae, but without assigning them to any subfamily. Therefore, at the beginning of the 20^th^ century, named species relevant to *Sadocus* were placed in both *Sadocus*, comprising two species (*S. polyacanthus* and *S. guttatus*) and *Lycomedes*, with five species (*L. asperatus*, *L. bicornis*, *L. calcitrosus*, *L. funestus* and *L. planiceps*).

Roewer (1913) designated *L. asperatus* as the type species of *Lycomedes*, and placed it in the Pachylinae. In that same work, he: (i) proposed the synonymy of *G. subsimilis* and *L. calcitrosus* with *L. asperatus*; (ii) described *Lycomedes calcar*; (iii) placed *Sadocus* in the Gonyleptinae and described *S. dilatatus* and *S. conspicillatus*. In his large work of 1923, Roewer proposed *Lycomedicus* as a replacement name for *Lycomedes*, which was preoccupied, and described as new the genus *Eubalta*.

Mello-Leitão (1937) described the genus *Carampangue* for his new species *C. ingens*, placing it in the Pachylinae, and in 1943, he described *C. nigronotatum*. In that same year, Roewer (1943) described the monotypic genus *Jighas* for the new species *J. vastus*, and placed it in the Pachylinae.

In 1945, Mello-Leitão described *Carampangue allermayeri*, and in the next year (1946), he described *Araucanoleptes* for the new species *A. exceptionalis*, and placed it in the Gonyleptinae. A few years later, Mello-Leitão (1949) synonymized *J. vastus* with the older *Carampangue ingens*. In that same year, Soares and Soares (1949) described *Lycomedicus brasiliensis*, and later, H. Soares (1968) transferred *Sadocus dilatatus* to *Lycomedicus*. During the next 30 years, only few catalogues (Cekalovic 1968, 1976, 1985) mentioned the species related to *Sadocus*. 154 years after its first description, Acosta (1996) studied the collection of type material of Pachylinae described by Roewer and found differences between Guérin-Méneville’s (1844) description of *L. planiceps* and the redescription by Roewer (1913).

At the beginning of the 21^st^ century (prior to this study), species relevant to *Sadocus* were placed in the following genera and subfamilies: the monotypic *Araucanoleptes* (Gonyleptinae); *Carampangue* (Pachylinae), with three species (*C. allermayeri*, *C. ingens* and *C. nigronotatum*); *Lycomedicus* (Pachylinae), with seven species (*L. asperatus*, *L. bicornis*, *L. brasiliensis*, *L. calcar*, *L. dilatatus*, *L. funestus* and *L. planiceps*); and *Sadocus* (Gonyleptinae), with three species (*S. conspicillatus*, *S. guttatus*, and *S. polyacanthus*).

Kury (2003), in his complete catalogue of New World Laniatores, proposed the synonymy of *Lycomedicus*, *Carampangue*, and *Araucanoleptes* with *Sadocus*. Hence, *Sadocus* comprised 14 species (actually, there are entries for 15 species, but that of *S. subsimilis* is clearly a mistake, which should be listed as a junior synonym under *S. asperatus*). Finally, Pessoa-Silva et al. (2020), transferred *S. planiceps* to *Eubalta*. *Sadocus* hitherto was composed of 13 species (Kury et al. 2020b). In the present publication, we accept only seven species of *Sadocus* as valid.

## Materials and methods

Material examined belongs to the following institutions (curators in parentheses) listed below:

**AMNH**American Museum of Natural History, New York, USA (L. Prendini);

**NHM**The Natural History Museum, London, England (J. Beccaloni);

**CAS**California Academy of Sciences (Entomology), San Francisco, California, USA (L. Esposito);

**MCZ**Museum of Comparative Zoology, Cambridge, Massachusetts, USA (G. Giribet);

**MNHN**Muséum National d’Histoire Naturelle, Paris, France (M. Judson);

**MNRJ**Museu Nacional, Universidade Federal do Rio de Janeiro, Rio de Janeiro, Brazil (A.B. Kury);

**MZSP**Museu de Zoologia, Universidade de São Paulo, São Paulo, Brazil (R. Pinto-da-Rocha). CGPC = Carlos Nicolau Gofferjé Private Collection was transferred to MZSP;

**SMF**Senckenberg Research Institute and Museum, Frankfurt am Main, Germany (P. Jäger);

**UFMG**Universidade Federal de Minas Gerais, Belo Horizonte, Brazil (A.J. Santos);

**URMU** Museo Nacional de Historia Natural de Montevideo, Montevideo, Uruguay (M. Simó);

**ZMB**Museum für Naturkunde Leibniz-Institut für Evolutions- und Biodiversitätsforschung, Berlin, Germany (J. Dunlop);

**ZMUC**Zoologisk Museum Universität København, Copenhagen, Denmark (N. Scharff).

The following abbreviations are used throughout the text, including synonymic listings:

**cat** catalogue;

**cit** citation;

**coll** collected;

**desc** description;

**eco** ecology;

**rdesc** redescription;

**syst** systematic discussion.

In the examined material:

**fe** female;

**ma** male;

**juv** juvenile;

**MS A–E** penis ventral plate pairs of macrosetae A–E.

[**X(y)**] where X is the character number and y, the character state.

The topological nomenclature follows Acosta et al. (2007), nomenclature of integumentary ornamentation of dorsal scutum and legs, dorsal scutum outline and ventral plate penial macrosetae follows DaSilva and Gnaspini (2010), Kury and Medrano (2016) and Kury and Villarreal (2015), respectively. Nomenclature of ovipositor morphology generally follows Townsend et al. (2015). We adopted the orientation of the captured images to reference the ovipositor lobes, because we had no topological reference after detaching it. It is unlike the penis, which has a sclerotized ventral feature, thus being easily referenced topologically. In *Sadocus*, we realized that leg IV is twisted retro-laterad from the trochanter (gradually untwisting along the femur), rendering the otherwise prolateral structures as dorsal (Fig. 3A, E). To standardize the topological nomenclature, we opted to consider (and call) those as prolateral, despite being functionally dorsal (in situ). We illustrated the external morphology using a stereomicroscope with a camera lucida and the material immersed in 70% ethanol. We prepared male and female genitalia according to Pinto-da-Rocha (1997) to take pictures using a scanning electron microscope (SEM) or to illustrate using a compound microscope with a camera lucida. The generic characteristics are not repeated in the specific (re)descriptions. Only characters differing from those of the males are listed in the female (re)descriptions. The variation on the number of tubercles on the dorsal scutum and other parts of the body or legs were included in the intraspecific variation. The color descriptions are based on specimens preserved in 70% ethanol and living photograph examples presented in the section “variation in males (or females)” under each species. Many species of *Sadocus* present a white patch on the body, commonly known as a dry-mark (Kury in DaSilva and Gnaspini 2009). It is an external serose layer of the cuticle that often forms white patches/shapes. Distribution maps for *Sadocus* species were prepared using QGIS 3.10 (QGIS.org 2019). The identification key is only for males. Synonymic listings follow Kury’s catalogue (2003), to which we add the category of its content between parentheses (see abbreviations section above). All measurements are in millimeters. We followed the view of Kury et al. (2020a) regarding the use of the correct inflection of specific epithets that are adjectives throughout the article to avoid inviting further confusion for the reader. Therefore, despite Kury himself (2003) proposing the combination *Sadocus funestis* (Butler, 1874), we use *Sadocus funestus* (Butler, 1874) as Kury et al. (2020b) in all sections (except for synonymic listing), including the historical aspects of *Sadocus*.

We chose the outgroups based on available hypotheses including Pachylinae, such as Pinto-da-Rocha et al. (2014), Hara et al. (2012) and Hara (2016). We rooted the tree in Stygnidae (*Stygnus polyacanthus* (Mello-Leitão, 1923)) based on Kury (1994). We added other taxa to account for the morphological diversity of Pachylinae sensu stricto and its sister group.

### List of outgroups analyzed, with respective vouchers

*Acanthopachylus aculeatus* (Kirby, 1819) (Gonyleptidae: Pachylinae) (MZSP 76419)

*Acanthoprocta conica* Maury, 1991 (Gonyleptidae: Pachylinae) (AMNH)

*Goniosoma varium* Perty, 1833 (Gonyleptidae: Goniosomatinae) (MZSP 76421)

*Gonyleptes horridus* Kirby, 1819 (Gonyleptidae: Gonyleptinae) (MZSP 59820)

*Metagyndes pulchella* (Loman, 1899) (Gonyleptidae: Pachylinae) (AMNH)

*Nanophareus polyhastatus* Hara, 2016 (Gonyleptidae: Pachylinae) (AMNH)

*Neogonyleptes docilis* (Butler, 1876) (Gonyleptidae: Pachylinae) (AMNH)

*Neogonyleptes karschii* (Sørensen, 1902) (Gonyleptidae: Pachylinae) (AMNH)

*Pachylus chilensis* (Gray, 1833) (Gonyleptidae: Pachylinae) (AMNH)

*Pachyloides thorellii* Holmberg, 1878 (Gonyleptidae: Pachylinae) (MZSP 59880)

*Roeweria bittencourti* Mello-Leitão, 1923 (Gonyleptidae: Roeweriinae) (MZSP 76420)

*Stygnus polyacanthus* (Mello-Leitão, 1923) (Stygnidae: Stygninae) (MZSP 59951)

We include records of distribution in maps only for vials with males. We used LibreOffice Calc to edit the character matrix and TNT 1.0 (Goloboff, Farris and Nixon 2008) to perform an implicit enumeration search under parsimony using equal weights. No character was ordered. We calculated Absolute and Relative Bremer support (Bremer 1994) to evaluate the support of clades using the Bremer Support Script for TNT 1.0 written by Pablo Goloboff (available at http://tnt.insectmuseum.org/index.php/Scripts/bremer). We used Winclada 1.00.08 (Nixon 1999) to edit the tree under ACCTRAN optimization and the notation of taxon+ proposed by Amorim (1982).

## Results and discussion

### Cladistic analysis

**Figure 1.**
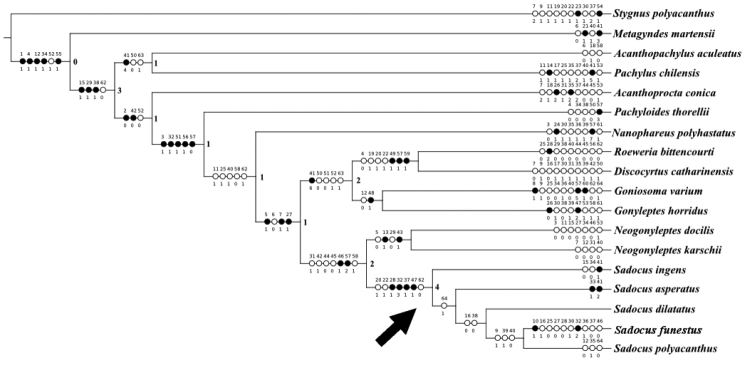
The single most parsimonious tree retrieved in the cladistic analysis representing *Sadocus* relationships (182 steps, L = 182; C.I. = 45; R.I. = 53). Number at the nodes are Goodman-Bremer support. Black circles indicate unique transformations, while white circles indicate homoplastic transformations, the arrow indicates the position of the genus *Sadocus*. The character number is above each circle, and the character state is below. The characters are optimized in ACCTRAN. The character and character states are given in Table 1, and the data matrix is in Table 2.

To test the monophyly of *Sadocus*, we used a matrix of morphological characters composed of 18 taxa (13 outgroups and five ingroups) and 64 characters (Table 1). The 64 characters are distributed as follows: 18 from dorsal scutum, four from free tergites, one from the chelicera, 23 from male legs, 17 from male genitalia, and one from coloration. We only included the valid *Sadocus* species with known males, as most of the characters are based on armature of male leg IV and penis. The cladistic analysis resulted in a single most parsimonious tree (182 steps, L = 182; C.I. = 45; R.I. = 53, Fig. 1).

**Table 1.** List of character and character states used in the cladistic analysis. All characters of legs and genitalia refer to male.

	Character	State
1	(DS) Ocularium (Hara 2016):	0. Divided, each eye placed onto different elevations; 1. Single.
2	(DS) Ocularium, unpaired armature:	0. Absent; 1. Present.
3	(DS) Ocularium, paired armature:	0. Absent; 1. Present.
4	(DS) Anterior margin of carapace, frontal hump	0. Inconspicuous; i.e., straight from ocularium to anterior margin of DS in lateral view 1. Conspicuous; i.e., clear elevation from ocularium to anterior margin of DS in lateral view
5	(DS) Mesotergum: placement of the maximum width	0. Maximum width in the middle of the mesotergum; 1. Maximum width placed posteriorly to the middle of mesotergum
6	(DS) Dorsal scutum length and width ratio	0. Wider than long; 1. Longer than wide
7	(DS) Posterior margin, shape	0. Straight; 1. Concave; 2. Convex
8	(DS) Area I, state of fusion	0. Divided in right and left halves by a longitudinal groove between scutal areas I – II (even though the groove of area II slightly invades area I) 1. Divided in right and left halves by invasion of scutal area II into middle of scutal area I.
9	(DS) Scutal area I,paramedian armature (Hara 2016)	0. Absent or with similar sized granules; 1. With a pair of tubercles
10	(DS) Scutal area II, paramedian paired armature (Hara 2016)	0. Absent or with similar sized granules; 1. With a pair of tubercles
11	(DS) Scutal area III, paramedian paired armature (Hara 2016)	0. Absent or with similar sized granules; 1. With a paramedian pair of tubercles
12	(DS) Scutal area IV, presence	0. Absent; 1. Present.
13	(DS) Scutal area IV, degree of division	0. Incompletely divided; i.e., interrupted scutal groove IV; 1. Completely divided
14	(DS) Scutal area IV, paramedian paired armature	0. Absent; 1. Present.
15	(DS) Lateral margin, type of integumentary ornamentation	0. Covered with granules; 1. With tubercles, sometimes clustered
16	(DS) Lateral margin, type of armature	0. Large tubercles or apophyses; 1. similar sized tubercles
17	(DS) Posterior margin of the DS, paramedian armature	0. Absent; 1. Present.
18	(DS) Posterior margin of the DS, central unpaired armature	0. Absent; 1. Present.
19	(DS) Free tergites I, paramedian armature	0. Absent; 1. Present.
20	(DS) Free tergites II, paramedian armature	0. Absent; 1. Present.
21	(DS) Free tergites II, unpaired armature	0. Absent; 1. Present.
22	(DS) Free tergites III, paramedian paired armature	0. Absent; 1. Present.
23	(Chelicerae) Chelicerae, sexual dimorphism (#30, Hara 2016)	0. Isomorphic in both sexes 1. Large in male
24	(Pedipalp) Tibia, type of retro-lateral apical seta	0. Single; 1. Bifid
25	(Leg) Coxa IV, branch of the prodorsal apophysis	0. Single; 1. Bifid
26	(Leg) Coxa IV, insertion of the prodorsal apical apophysis in relation to the DS main axis (Hara et al. 2012)	0. Almost transversal; i.e., almost 90 degrees in relation to DS main axis; 1. Oblique; i.e., more than 120 degrees in relation to DS main axis; 2. Parallel to femur IV
27	(Leg) Coxa IV, retro-apical apophysis:	0. Absent; 1. Present.
28	(Leg) Leg IV, torsion that begins at the trochanter and ends at the patella	0. Untwisted; 1. Strongly twisted; i.e., prolateral features becoming dorsal in situ and gradually untwisting towards patella; 2. Strongly twisted from coxa IV towards the patella (Fig. 3A, E).
29	(Leg) Trochanter IV, Prolateral basal apophysis (# 30 Hara & Pinto–da–Rocha 2010)	0. Absent; 1. Present.
30	(Leg) Trochanter IV, dorso-median subapical apophysis	0. Absent; 1. Present.
31	(Leg) Trochanter IV, prodorsal apical apophysis	0. Absent; 1. Present.
32	(Leg) Trochanter IV, type of prodorsal apical apophysis	0. As a wart; 1. As a hook-like pointed apophysis of large base, smoothly becoming pointed apically; 2. As a moderate size blunt cone; 3. As a finger shaped, robust apophysis, basal half of ca. uniform diameter
33	(Leg) Trochanter IV, retro-dorsal apical apophysis (# 29 Hara & Pinto–da–Rocha 2010)	0. Absent; 1. Present.
34	(Leg) Trochanter IV, retro-apical armature (Hara & PdR 2010)	0. Absent; 1. Present.
35	(Leg) Trochanter IV, type of retro-apical armature	0. Pointed tubercle; 1. Moderate apophysis (ca. a quarter of podomere width); 2. Huge apophysis (as long as podomere width).
36	(Leg) Trochanter IV, proapical apophysis	0. Absent; 1. Present.
37	(Leg) Trochanter IV, length-width ratio (modified from Hara 2016)	0. As long as wide; 1. Twice longer than wide; 2. Wider than long.
38	(Leg) Femur IV, curvature in dorsal view	0. Straight; 1. Sinuous
39	(Leg) Femur IV, size of granules on retro-lateral row	0. Similar sized granules; 1. Tubercles, twice the size of granules.
40	(Leg) Femur IV, spiniform apophyses on basal half of the retro-lateral row of granules	0. Absent; 1. Present.
41	(Leg) Femur IV, the pattern of apophyses distribution at the ⅔ basal region of the retro-lateral row	0. Just an apophysis in the basal ⅓; 1. Growing from the median region to the basal region; 2. Very high apophyses alternating with low apophyses; 3. Apophyses distributed in the median region; 4. A basal apophysis and one or more in the distal ⅔; 5. An average apophysis; 6. Decreasing from the median region to the basal region.
42	(Leg) Patella IV, ventral row of granules	0. Similar sized granules; 1. Granules becoming tubercles or spines.
43	(Leg) Tibia IV, ventro-basal long spine	0. Absent; 1. Present.
44	(Leg) Tibia IV, size of granules on retro-ventral row	0. Tubercles of similar sizes; 1. Granules increasing in size apically, becoming tubercles.
45	(Leg) Tibia IV, proventral row of tubercles size	0. Tubercles of similar sizes; 1. Larger tubercles, which grows in size apically.
46	(Leg) Tibia IV, size of granules on ventral row	0. Similar sized granules; 1. Increasing in size apically.
47	(Penis) Ventral plate, shape of the distal margin	0. Straight; 1. Slightly concave; 2. Very concave, forming a “U”.
48	(Penis) Ventral plate, basal lobes	0. Inconspicuous; 1. Conspicuous.
49	(Penis) Ventral plate, plate format	0. Rectangular; 1. Hexagonal.
50	(Penis) Ventral plate, number of MS C	0. Three pairs; 1. Four pairs or more.
51	(Penis) Glans, dorsal prominence in the distal region of the sac	0. Absent; 1. Present.
52	(Penis) Glans, sac texture	0. Smooth and turgid; 1. Wrinkled.
53	(Penis) Glans, latero-apical region	0. Without projections; 1. With projections covering part or all of the pedestal in lateral view.
54	(Penis) Glans, dorsal process	0. Absent; 1. Present.
55	(Penis) Glans, ventral process	0. Absent; 1. Present.
56	(Penis) Ventral process, presence of stem	0. Absent; 1. Present.
57	(Penis) Ventral process, apex shape	0. As a flabellum ; 1. Tapered at the apex and rolled; 2. Flattened circular; 3. Flattened quadrangular; 4. Fringed triangular; 5. Large rectangular; 6. Rectangular bifid; 7. Rectangular with pointed projections.
58	(Penis) Stylus, ventral process length ratio	0. Stylus shorter than ventral process; 1. Stylus longer than ventral process.
59	(Penis) Stylus, apical lateral projections	0. Absent; 1. Present.
60	(Penis) Stylus, apex shape (DaSilva and Gnaspini 2010)	0. Rounded; 1. With an apical back beak.
61	(Penis) Stylus, trichomes on median apical region	0. Absent; 1. Present.
62	(Penis) Insertion on the glans in lateral view	0. Ventral; 1. Median
63	(Penis) Trunk of the penis, subapical region	0. Truncated; 1. Projected on the glans
64	(Color) Carapace, presence of dry-mark	0. Absent; 1. Present.

According to the retrieved tree, *Sadocus* is not monophyletic, as it excludes *Sadocus brasiliensis* (Soares & Soares, 1949). Acosta (2020) first mentioned that *S. brasiliensis* may not belong to this genus based on the overall distribution of the other *Sadocus* species. That suspicion is corroborated here, and we propose its synonymy with the Brazilian *Discocyrtus catharinensis* (Mello-Leitão, 1923) (see taxonomic notes in this species’ entry). In turn, the close relationship of *D. catharinensis* with *Roeweria bittencourti* (Roeweriinae Brazilian species) is supported by seven synapomorphies, three of them exclusive: hexagonal shape of the penial ventral plate [49(1)]; penial ventral process apex tapering distally, becoming rolled [57(1)]; and penial stylus with latero-apical projections [59(1)]. *Discocyrtus catharinensis* is probably a Roeweriinae especially based on the shape of the penial ventral plate as well as the overall penial morphology. However, we refrained further taxonomic actions as Roeweriinae diversity grows further fueled by the dismemberment of *Discocyrtus*, which is currently under revision (Kury and Carvalho 2016; Carvalho and Kury 2018, 2021).

**Table 2.** Matrix of character states for the cladistic analysis of the *Sadocus* (Gonyleptidae: Pachylinae).

*Stygnus polyacanthus*	0	1	0	0	0	1	2	0	1	0	1	0	-	-	0	1	0	0	1	1	0	1	1	0	0	1	0	0	0	1	0	-	0	0	-	0	2	0	0	0	-	1	0	1	1	0	0	0	0	1	0	0	0	1	0	-	-	-	-	0	0	1	0	0
*Acanthopachylus aculeatus*	1	1	0	1	0	0	0	0	0	0	0	1	0	0	1	1	0	1	0	0	0	0	0	0	0	1	0	0	1	0	0	-	0	1	0	0	0	1	0	0	4	1	0	1	1	0	0	0	0	0	0	1	0	0	1	0	4	0	0	0	0	0	1	0
*Acanthoprocta conica*	1	0	0	1	0	1	2	0	0	0	0	1	0	0	1	1	0	1	0	0	0	0	0	0	0	2	0	0	1	0	1	0	0	1	2	0	2	1	0	0	-	0	0	0	0	0	0	0	0	1	0	0	1	0	1	0	4	1	0	0	0	0	0	0
*Goniosoma varium*	1	0	1	1	1	0	1	1	1	0	1	0	-	-	1	1	0	0	0	0	0	0	0	0	0	1	1	0	1	0	0	-	0	0	-	1	0	1	0	0	-	0	0	1	1	0	0	1	0	0	0	1	0	0	1	1	5	0	0	1	0	0	1	1
*Gonyleptes horridus*	1	0	1	1	1	0	1	0	0	0	1	0	-	-	1	1	0	0	0	0	0	0	0	0	1	0	1	0	1	1	0	-	0	1	0	0	0	0	1	1	6	0	0	1	1	0	2	1	0	0	0	1	1	0	1	1	0	1	0	0	1	1	1	0
*Metagyndes martensii*	1	1	0	1	0	0	0	0	0	0	0	1	0	0	0	1	0	0	0	0	1	0	0	0	0	1	0	0	0	0	0	-	0	1	0	0	0	0	0	1	3	1	0	1	1	0	0	0	0	1	0	1	0	0	1	0	4	1	0	0	0	1	0	0
*Nanophareus polyhastatus*	1	0	0	1	0	1	0	0	0	0	1	1	0	0	1	1	0	0	0	0	0	0	0	1	1	1	0	0	1	1	0	-	0	1	1	1	0	1	1	1	0	0	0	1	1	0	0	0	0	1	1	0	0	0	1	1	7	0	0	0	1	1	0	0
*Neogonyleptes docilis*	1	0	0	1	0	0	1	0	0	0	0	1	1	0	0	1	0	0	0	0	0	0	0	0	1	1	0	0	0	0	1	1	0	0	-	0	0	1	0	1	0	1	1	0	0	0	0	0	0	1	1	0	1	0	1	1	2	1	0	0	0	1	0	0
*Neogonyleptes karschii*	1	0	1	1	0	0	0	0	0	0	1	0	-	-	1	1	0	0	0	0	0	0	0	0	1	1	1	0	0	0	0	1	0	1	0	0	0	1	0	0	-	1	1	0	0	1	0	0	0	1	1	0	0	0	1	1	2	1	0	0	0	1	0	0
*Pachyloides thorellii*	1	0	1	0	0	1	0	0	0	0	0	1	0	0	1	1	0	0	0	0	0	0	0	0	0	1	0	0	1	0	0	-	0	0	-	?	0	0	0	0	-	0	0	1	1	0	0	0	0	0	1	0	0	0	1	1	3	1	0	0	0	0	0	0
*Pachylus chilensis*	1	1	0	1	0	1	0	0	0	0	1	1	0	1	1	1	1	0	0	0	0	0	0	0	1	1	0	0	1	0	0	-	0	1	1	0	2	1	0	1	5	1	0	1	1	0	0	0	0	0	0	1	1	0	1	0	4	1	0	0	0	0	1	0
*Roeweria bittencourti*	1	0	1	0	1	0	1	0	0	0	1	1	0	0	1	1	0	0	1	1	0	1	0	0	0	1	1	2	0	0	0	-	0	1	0	0	0	0	0	0	-	0	0	0	0	0	0	0	1	0	0	1	0	0	1	0	-	-	1	0	0	0	1	0
*Discocyrtus catharinensis*	1	0	1	0	1	0	0	0	1	0	1	1	0	0	1	0	1	0	1	1	0	1	0	0	1	1	1	0	1	1	1	1	0	1	1	0	0	1	1	1	6	1	0	1	1	0	0	0	1	1	0	1	0	0	1	1	1	0	1	0	0	1	1	0
*Sadocus asperatus*	1	0	1	1	1	0	1	0	0	0	1	1	0	0	1	1	0	0	0	1	0	1	0	0	1	1	1	1	1	0	1	3	1	1	0	0	1	1	0	1	2	1	0	0	0	1	1	0	0	1	1	0	0	0	1	1	2	1	0	0	0	0	0	1
*Sadocus dilatatus*	1	0	1	1	1	0	1	0	0	0	1	1	0	0	1	0	0	0	0	1	0	1	0	0	1	1	1	1	1	0	1	3	0	1	0	0	1	0	0	1	0	1	0	0	0	1	1	0	0	1	1	0	0	0	1	1	2	1	0	0	0	0	0	1
*Sadocus funestus*	1	0	1	1	1	0	1	0	1	1	1	1	0	0	1	1	0	0	0	1	0	1	0	0	0	1	0	0	1	1	1	2	0	1	0	1	0	0	1	0	-	1	0	0	0	0	1	0	0	1	1	0	0	0	1	1	2	1	0	0	0	0	0	1
*Sadocus ingens*	1	0	1	1	1	0	1	0	0	0	1	1	0	0	0	1	0	0	0	1	0	1	0	0	1	1	1	1	1	0	1	3	0	0	-	0	1	1	0	1	1	1	0	0	0	1	1	0	0	1	1	0	0	0	1	1	2	1	0	0	0	0	0	0
*Sadocus polyacanthus*	1	0	1	1	1	0	1	0	1	0	1	0	-	-	1	0	0	0	0	1	0	1	0	0	1	1	1	1	1	0	1	3	0	1	1	0	1	0	1	0	-	1	0	0	0	1	1	0	0	1	1	0	0	0	1	1	2	1	0	0	0	0	0	0

Once we settled the issue related to *S. brasiliensis*, we propose a new concept of *Sadocus*. Under the new definition, *Sadocus* is monophyletic and supported by seven synapomorphies, four of which are exclusive: leg IV twisted from the trochanter to patella [28(0)]; trochanter IV with a finger shaped, robust prodorsal apical apophysis, its basal half of ca. uniform diameter [32(3)], trochanter IV twice longer than wide [37(1)] (modified from Hara 2016); and penis ventral plate with slightly concave distal margin [47(1)]. *Sadocus* is also the best supported clade of the analysis, with a high Goodman-Bremer support (4).

So far, *Sadocus* (represented especially by *S. polyacanthus*, its type species) has often been used in cladistic analysis as outgroups (Hara et al. 2012; Hara 2016) or as an ingroup taxon of a more comprehensive analysis testing monophyly of Gonyleptidae or Gonyleptoidea (Pinto-da-Rocha et al. 2014; Benavides et al. 2021). According to the analyses based on morphological characters (Hara et al. 2012; Hara 2016), *Sadocus* is nestled in a clade mainly composed of Brazilian species. However, we have to stress that the clade with *Sadocus* in Hara’s analyses is not well supported (Bremer support: 1), its sole homoplastic synapomorphy being the proventral apical armature of tibia IV as a tubercle. In the present analysis, we have a roughly similar outcome, as the clade including *Sadocus* (*Sadocus* + *Neogonyleptes*) is sister group to a clade composed of solely Brazilian species. This outcome differs considerably from Pinto-da-Rocha et al. (2014) or Benavides et al. (2021): in those analyses based on molecular data, *Sadocus* is often retrieved closely related to Chilean Pachylinae genera. Regarding this, Pinto-da-Rocha et al. (2014) indicate that *Sadocus* is in a clade with other Chilean species (*Neogonyleptes karschii* and *Tumbesia aculeata*), which in turn is sister group to Pachylinae sensu stricto. Benavides et al. (2021) also corroborates a close relationship of *Sadocus* with Chilean genera.

The sister taxon closest to *Sadocus* is also an unsettled issue, mainly because different taxa are employed in those analyses. In the present analysis, *Sadocus* sister group is the Chilean genus *Neogonyleptes*, supported by seven synapomophies, two of them exclusive: ventral row of granules increasing in size apically on tibia IV [46(1)]; and apex of glans ventral process flattened circle shaped [57(2)]. This sister group relationship is similar to Pinto-da-Rocha et al. (2014) sampling wise. On the other hand, Benavides et al. (2021) did not include *Neogonyleptes* in their analysis, and the Chilean *Eubalta planiceps* is the sister taxon to *Sadocus*. It is interesting to note that *Sadocus* sister taxon is strongly affected by the Chilean Pachylinae sampling not belonging to Pachylinae sensu stricto.

The main goals of this study were to revise and to test the monophyly of *Sadocus*, a hundred-year-old genus, with a convoluted taxonomic history. We believe that we succeeded in those, and the present study is an important step towards the understanding the evolution of the genus. Considering all the evidence (including the taxonomic history), *Sadocus* seems to be related to the Chilean-Argentinean Pachylinae. We understand that *Sadocus* relationship within Gonyleptidae is still an unsettled issue that deserves further investigation. As a mean to tackle that, we can suggest the inclusion of more Chilean Pachylinae genera (especially those already used in previous analyses and not belonging to Pachylinae sensu stricto) and Brazilian species as well, such as DRMN (Carvalho and Kury 2018) and K92 (Kury 1992). The latter suggestion is because of the Brazilian clade closest to Chilean Pachylinae clade depicted by Pinto-da-Rocha et al. (2014) and Benavides et al. (2021).

### Taxonomic accounts

#### Gonyleptidae Sundevall, 1833

**Pachylinae Sørensen, 1884**

##### Sadocus

Taxon classificationAnimaliaOpilionesGonyleptidae

Sørensen, 1886

64992B26-2678-5565-9C34-FB3B4DBA0E0D

Gonyleptes [part]: Gervais, 1842: 2 [rdesc]; 1844: 105 [rdesc]; 1847: 576–577 [cit]; 1849: 21, 24–26 [desc, rdesc]; Butler 1873: 113–114 [cat]; 1876: 153 [desc].
Discocyrtus [part]: Loman, 1899: 6 [desc].
Sadocus Sørensen, 1886: 85 [desc]; 1902: 14–20 [rdesc]; Hogg 1913: 48 [cit]; Roewer 1913: 244–245 [cit, key]; 1923: 492 [cit, key]; Mello-Leitão 1923: 190 [cit]; 1926: 31 [key]; Roewer 1930: 381 [key, cit]; Mello-Leitão 1931a: 136 [cit]; 1932: 348 [rdesc]; 1935: 105 [key]; Canals 1936: 70 [cat]; Kästner 1937: 389 [desc]; Roewer 1938: 02 [cit]; Mello-Leitão 1939: 625 [cit]; B. Soares 1944a: 166 [syst]; Soares and Soares 1949: 211 [rdesc]; Ringuelet 1955a [cit]: 438; Ringuelet 1959: 409 [rdesc]; Roewer 1961: 102 [cat]; Cekalovic 1985: 14 [cat]; Kury 2003: 191 [cat]; Hara et al. 2012: 38–39 [syst]; Pinto-da-Rocha et al. 2012: 61 [cit]; Pinto-da-Rocha et al. 2014: 18 [syst]; Hara 2016: 106–109 [syst]; Pérez-Schultheiss et al. 2019, 4, 12–15 [cit]; Kury et al. 2020a: 5[cit]; Acosta 2020 [cit]; Kury et al. 2020b [cat]. (Type species Sadocus vitellinosulcatusSørensen 1886, by monotypy).
Lycomedes Sørensen, 1902: 17 [desc]; Roewer 1913: 126–127 [rdesc, key]; Mello-Leitão 1926: 31 [key]; Muñoz-Cuevas 1973: 226–228 [cit]. (Type species Gonyleptes asperatus Gervais, 1847, by subsequent designation by Roewer 1913).
Lycomedicus Roewer, 1923: 442 (nom. nov. for Lycomedes Sørensen, 1902, rdesc); 1925: 17 [cit]; 1929: 213 [cat]; Mello-Leitão; 1931b: 84 [cit]; 1932: 216 [rdesc]; 1935: 101 [cit]; Canals 1936: 69 [cat]; Roewer 1943: 28 [cit]; Ringuelet 1959: 329 [rdesc]; Cekalovic 1985: 18 [cat]. Synonym established by Kury 2003.
Lycomedius [lapsus]: Kästner, 1937: 389 [rdesc]; Strand 1942: 397 [cit].
Carampangue Mello-Leitão, 1937: 152 [desc]; 1945: 156 [cit]; 1949: 17 [syst]; Soares and Soares 1954: 241 [rdesc, cat]; Cekalovic 1985: 16 [cat]. (Type species Carampangue ingens Mello-Leitão, 1937 by monotypy). Synonymy established by Kury 2003.
Jighas Roewer, 1943: 28 [desc]; Mello-Leitão 1949: 17 [syst]. (Type species Jighas vastus Roewer, 1943 by monotypy). Synonymy established with Carampangue by Mello-Leitão 1949.
Araucanoleptes Mello-Leitão, 1946: 4 [desc]; Soares and Soares 1949: 160 [rdesc]. (Type species Araucanoleptes exceptionalis Mello-Leitão, 1946 by monotypy). Synonymy established by Kury 2003.
Arauconoleptes [*lapsus*]: Cekalovic, 1985: 12 [cat].

###### Type species.

*Sadocus vitellinosulcatus* Sørensen, 1886, by monotypy. Synonymized with *S. polyacanthus* by Sørensen (1902).

###### Other species included.

*S. asperatus* (Gervais, 1847), *S. dilatatus* Roewer, 1913, *S. funestus* (Butler, 1874), *S. ingens* (Mello-Leitão, 1937) and *S. polyacanthus* (Gervais, 1847).

###### Diagnosis.

*Sadocus* are large Pachylinae (dorsal scutum maximum length 5.5–13.8 mm) with paired spines on ocularium and prominent frontal hump on dorsal scutum anterior margin. Dorsal scutum shape types gamma triangular and gamma pyriform, its posterior margin concave. Dorsal scutum mid-bulge placed close to scutal groove IV (scutal groove III in *S. funestus*) and transversal (*S. funestus*, *S. ingens*, *S. polyacanthus*) or oblique (*S. asperatus*, *S. dilatatus*); free tergites II and III each with a pair of spines. Legs IV are twisted retro-laterad from the trochanter and gradually distorted along the femur and patella (except in *S. funestus*). Coxa IV bearing a long, large prodorsal apical apophysis and a short, retro-apical one (except in *S. funestus*, in which is lacking). Trochanter IV with a short, blunt prolateral sub-basal apophysis and a long, robust prodorsal apical one. Penis glans turgid and dorsally projected (with antero-lateral projections), with ventral process (half stylus length) and without dorsal process. General color (in living specimens) of the body and most parts of legs and ventral area dark brown, with lighter tones at the tips of podomeres. Yellowish to reddish tone in scutal area, scutal posterior margin, free tergites, part of legs and apophysis. Green on the arthrodial membranes between the free tergites.

###### Redescription.

**Male: *Dorsum*.** Anterior margin of carapace with a prominent median frontal hump (bell shaped in dorsal view). Ocularium with one pair of spines posterior to the eyes. Dorsal scutum type varying from gamma to gamma triangular and gamma pyriform, its posterior margin concave, mid-bulge slightly asymmetrical and displaced posteriorly, widest at the scutal groove IV (scutal groove III in *S. funestus*). The curvature of mid-bulge can be transversal (*S. funestus*, *S. ingens*, and *S. polyacanthus*) or oblique (*S. asperatus* and *S. dilatatus*). Four scutal areas (three in *S. polyacanthus*); scutal area I divided into right and left halves by a longitudinal median groove. Scutal area III with one pair of paramedian spiniform tubercles or spines. Two pairs of ozopores close to coxa II. Lateral margin of dorsal scutum with an external and internal rows of tubercles (the external row of slightly larger tubercles) (except *S. asperatus*, with granules covering most of the lateral margin of dorsal scutum and *S. ingens*, smooth or with only few granules). Posterior margin of dorsal scutum and free tergite I each with one paramedian pair of tubercles (except *S. funestus* and *S. polyacanthus*, unarmed). Free tergites II and III each with one paramedian pair of spines. ***Venter*.** Coxa I–IV granulate; coxa I with a median longitudinal row of granules increasing in size apically, becoming tubercles. ***Chelicerae*.** Isomorphic in males and females. Segment I with well-marked bulla. Segment II fixed finger and segment III toothed. ***Pedipalps*.** Trochanter dorsal face inflated; ventral face with one or two setiferous tubercles. Femur bearing sub-apical mesal seta; dorsal face with few granules; ventral face with one basal setiferous tubercle. Tibiae and tarsi dorsal and lateral faces with few minute granules and variable setation. ***Legs.*** Coxae I–III each with one prodorsal and one retro-dorsal spiniform tubercles, ventral faces granulate (except *S. polyacanthus*, coxa I with tubercles and others with setae). Coxa IV dorso-lateral face with sparsely distributed granules, ventral face entirely granulated, with one long, oblique, bifid prodorsal apical apophysis (transversal in *S. dilatatus*, uniramous in *S. funestus*), dorsal branch longest and curved ventrad and ventral branch short and blunt; and one ventro-apical retro-lateral spine. Trochanters I–III granulate. Leg IV twisted retro-laterad from the trochanter, gradually untwisting along the femur (except *S. funestus*, straight). Trochanter IV longer than wide; prolateral face with one short, conical, blunt sub-basal apophysis, and one robust, blunt dorso-apical apophysis. Femora I–IV with granules roughly organized in six longitudinal rows (prodorsal, retro-dorsal, pro- and retro-lateral, proventral and retro-ventral rows); femora I and II unarmed. Femur IV curved, with marked inner curvature on the distal half (*S. asperatus* and *S. ingens*) or almost straight (*S. dilatatus*, *S. funestus*, and *S. polyacanthus*). Patellae I–III granulate, unarmed; patella IV dorsal face granulate, ventral face tuberculate. Tibiae I–III granulate, unarmed (except *S. dilatatus* and *S. funestus*, tibia III dorsal face granulate with retro-ventral row of tubercles increasing in size apically). Tibia IV dorsal face granulate, ventral face with tubercles sparsely distributed. Metatarsi I–IV minute granulate, unarmed. Tarsus III and IV each with ventral process, tarsal claws smooth. ***Penis***. Ventral plate distal margin with slight (but conspicuous) to moderate concavity, two or three pairs of MS A, one pair of MS B or entirely absent, four or five pairs of MS C, one or two pairs of MS D, and one or two pairs of MS E. Glans sac tall, turgid, dorsally projected with antero-lateral projections, forming a sheath for the stylus. Glans without dorsal process; stylus inserted ventrally and smooth. Glans ventral process is short (half of stylus length), parallel to the stylus, apex curved ventrad, with a short semi-circular antero-lateral projection.

###### Geographic distribution

**Figure 2.**
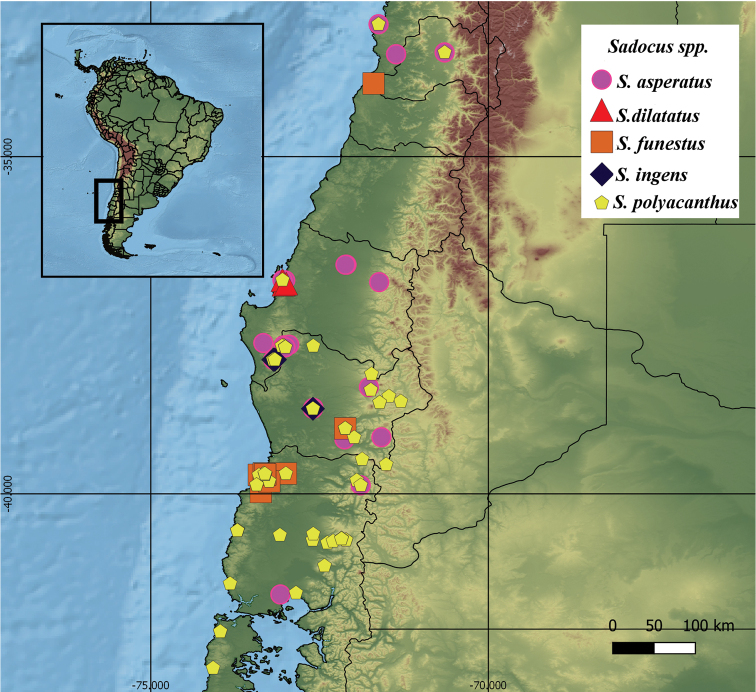
Geographical records of distribution of *Sadocus* species.

**(Fig. 2).** Central Chile, Región XIV Los Ríos; Metropolitan Region of Santiago; Región V Valparaíso; Región VIII Bio-Bío; Región IX Araucanía; and Region X Los Lagos. There are other localities mentioned in the literature besides the material studied here for *S. polyacanthus* in Neuquén (Argentina) and Magallanes, in the extreme south of Chile, however, we did not examine any material from there. The record of *Sadocus funestus* for Ecuador (Chimborazo, Riobamba) by Roewer (1913) is certainly a mislabeling because it does not agree with the known generic distribution (Cekalovic 1985; Kury 2003). Two species are widely distributed (*S. asperatus* and *S. polyacanthus*) and three others occur mainly in coastal mountains of Central Chile (*S. funestus*, *S. dilatatus*, and *S. ingens*).

### Key to males of *Sadocus* species

**Table:** 

1	Coxa IV with a bifid prodorsal apical apophysis (Fig. 3A, C) and one retro-ventral apical apophysis (Fig. 3A)	**2**
–	Coxa IV with an unbranched prodorsal apical apophysis (Fig. 5A, C), without retro-ventral apical apophysis (Fig. 5A)	***S. funestus***
2	Lateral margin of the dorsal scutum with a posterior large tubercle (Figs 4A, 7A)	3
–	Lateral margin of the dorsal scutum only with similar sized tubercles (Fig. 6C)	4
3	Dorsal scutum with four areas (Fig. 4A), femur IV with a long, retro-lateral sub-basal apophysis (Fig. 4D)	***S. dilatatus***
–	Dorsal scutum with three areas (Fig. 7A), femur IV retro-lateral face with spines of similar size (Fig. 7B)	***S. polyacanthus***
4	Trochanter IV dorso-apical face only with one prolateral apophysis of similar length as the podomere, strongly curved (in lateral view), pointing frontwards (Fig. 6C)	***S. ingens***
–	Trochanter IV dorso-apical face with two apophyses, both shorter than the podomere length, femur IV slightly curved (Fig. 3B, E)	***S. asperatus***

**Figure 3.**
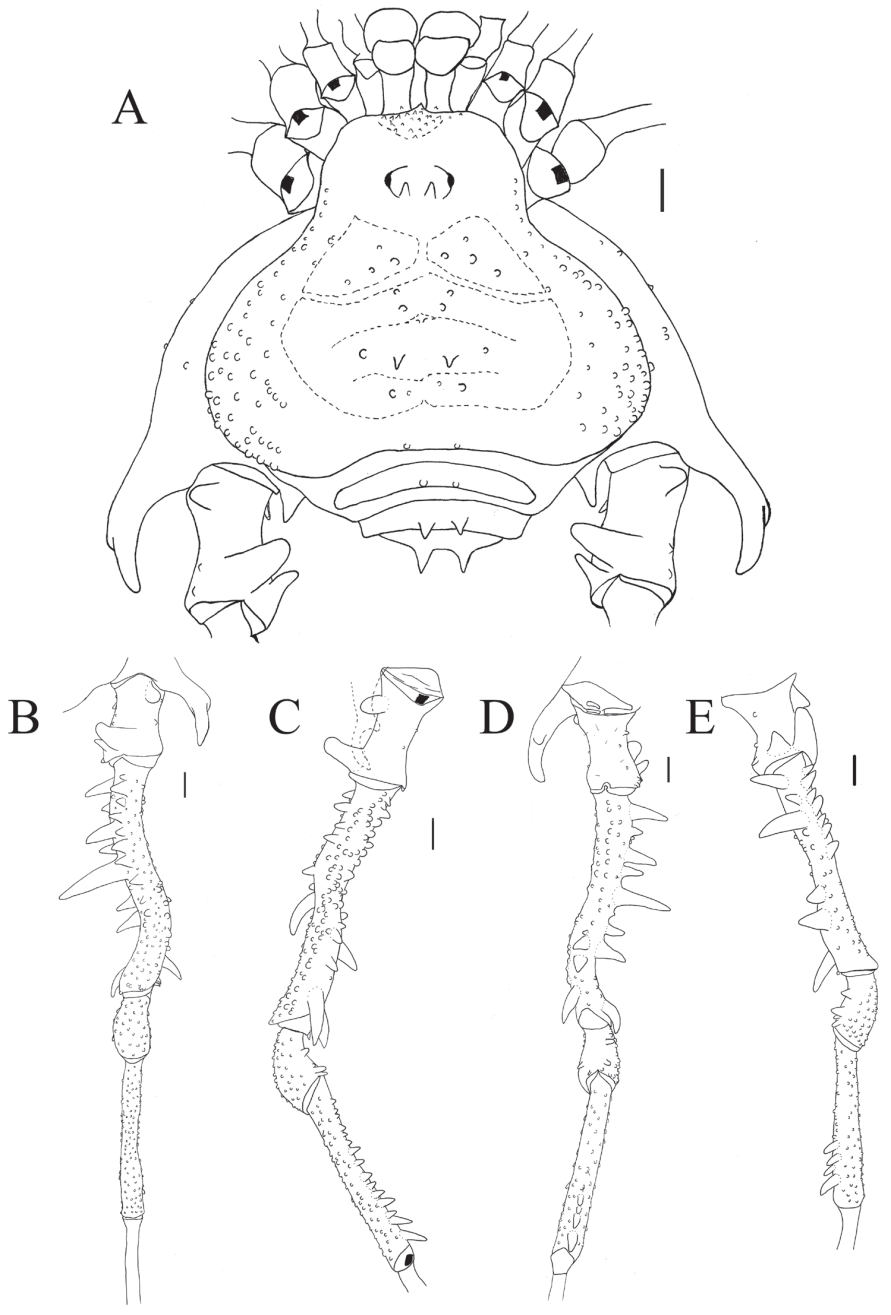
*Sadocus asperatus* (Gervais). Male (CAS 9055035) **A** Habitus, dorsal view **B–E** right leg IV, Trochanter–tibia IV **B** dorsal view **C** prolateral view **D** ventral view **E** retro-lateral view. Scale: 1 mm.

#### Sadocus asperatus

Taxon classificationAnimaliaOpilionesGonyleptidae

(Gervais, 1847)

F5D43E8E-278D-5AE2-8249-E292A2DB4ACF

Figures 2 , 3A–E , 8A, B , 9A–C, E , 11A, B , 12A–C

Gonyleptes asperatus Gervais, 1847: 577 [desc]; 1849: 26, pl. 1, fig 9 [rdesc]; Butler 1873: 113 [cat]; Sørensen 1902: 17 [syst]. Transferred to Lycomedes by Sørensen 1902. (MNHN, type lost, not examined) (RPR visited the museum but the curator was unable to find the type material)
Lycomedes asperatus : Sørensen, 1902: 17 [syst]; Roewer 1913: 127–130, fig 57 [key, rdesc].
Lycomedicus asperatus : Roewer, 1923: 442, fig 556 [rdesc]; 1925: 17 [cit]; 1929: 213 [cat]; Mello-Leitão 1931b: 84 [cit]; Canals 1936: 69 [cat]; Roewer 1938: 6 [cat]; Mello-Leitão 1939: 624 [cat]; Soares and Soares 1954: 270 [cat]; Ringuelet 1959: 329, fig 44 [rdesc]; H. Soares 1968: 264 [cit]; Cekalovic 1968: 8 [cat]; 1985: 18 [cat].
Sadocus asperatus : Kury, 2003: 191 [cat]; Kury et al. 2020b [cat].
Sadocus (?) subsimilis: Sørensen, 1902: 17 [cit]; Roewer 1913: 127–130, fig 57 [syst]. Synonymy with L. asperatus established by Roewer 1913.
Gonyleptes polyacanthoides Gervais, 1847: 577 [desc]; Butler 1873: 114 [cat]. Synonymy with G. subsimilis established by Butler 1873. (MNHN, type lost, material not examined) (RPR visited the Museum but the curator was unable to find the type material)
Gonyleptes subsimilis Gervais, 1849 [desc]: 25, pl. 1, fig 8; Butler 1873: 114 [cat]; Sørensen 1902: 6 [syst], 16 [syst]. Synonymy with Sadocus (?) subsimilis established by Sørensen 1902 (MNHN, type lost, material not examined) (RPR visited the museum but the curator was unable to find the type material)
Gonyleptes bicornis Gervais, 1849: 21, pl. 1, fig 4a–b [desc]; Butler 1873: 114 [cat]; Sørensen 1902: 19 [syst]. (type material depository unknown). syn. nov.
Lycomedes bicornis : Sørensen, 1902: 20, fig 4–4b [rdesc]; Roewer 1913: 136–137, fig 62 [rdesc].
Lycomedicus bicornis : Roewer, 1923: 445, fig 561 [cit]; Canals 1936: 69 [cat]; Soares and Soares 1954a: 270 [cat]; Cekalovic 1968: 7 [cat], 1985: 18 [cat].
Sadocus bicornis : Kury, 2003: 191 [cat]; Kury et al. 2020b [cat].
Discocyrtus calcitrosus Loman, 1899: 7, fig 5 [desc]; Sørensen 1902: 19 [syst]. (type ZMB 7837, ma holotype – examined by detailed photographs).
Lycomedes calcitrosus : Sørensen, 1902: 19 [syst]. Synonymy with L. asperatus established by Roewer 1913.
Lycomedicus calcitrosus : Moritz, 1971: 193 [cat].

##### Material examined.

Chile, date and collector unknown, 1 ma (MCZ 31267). Región Metropolitana de Santiago, *Santiago*, date or collector unknown, 6 ma, 3 fe (SMF 1369); Same, *El Canelo*, 16.I.1980, collector unknown, 2 ma (AMNH); Same, 16.I.1980, collector unknown, 1 ma, 1 fe (AMNH). Región de Valparaíso, collection date or collector unknown, 8 ma, 19 fe (SMF 5382). Región de Biobío, *Provincia de Concepción*, 22.I.1985, N.I. Platnick & O.F. Francke coll., 1 ma (AMNH); Same, Estero Nonguén (-36.831501, -73.008374), 13.III.1977, T. Cekalovic coll., 1 ma (MCZ 31275); Same, Cerro Caracol, Mirador Aleman (-36.834167, -73.047778), 15.IX.1968, T. Cekalovic coll., 1 ma, 3 fe (MZSP 9965); Same, *Provincia de Arauco*, Parque Nacional Nahuelbuta (-37.800000, -73.033333), 30.XI.2003, I. Avila, S. Ocares & D. Silva coll., 1 ma (CAS 9055050); Same, Parque Nacional Nahuelbuta (-37.8043374259, -73.0344813614, date or collector unknown, 1 ma, 1 fe (CAS 9052227); Same, Parque Nacional Nahuelbuta (-37.827500, -73.009722), 9.XII.2010, F. Marques, F. Cadiz & F. Carbayo coll., 5 ma (MZSP 36839); Same, Parque Nacional Nahuelbuta, (-37.827500, -73.009722), 9.XII.2010, F. Marques, F. Cadiz & F. Carbayo coll., 6 ma, 1 fe (MZSP 36840); Same, Cayucupil, Pichinahuel, 23.XII.1976, collector unknown, 1 ma (AMNH); Same (-37.766667, -73.333333), 9.II.1965, T. Cekalovic coll., 1 ma (MZSP 27695). Región de Nuble, *Provincia Diguillín*, Chillán, Las trancas Nuble, XII.1976, collector unknown, 1 ma, 3 fe (AMNH); Same, ?.XII.1976, collector unknown, 1 ma, 2 fe (AMNH); Same, 11–17.I.1983, L.E. Pena coll., 1 ma (AMNH); Same, XII.1974, collector unknown, 1 ma, 3 fe (AMNH); Same, Las Trancas, (-36.600, -72.117), I.1967, L. Peña coll, 1 ma (MCZ 38272); Same, Los Lleuques (-36.85800, -71.61700), 11–7.V.1975, G. Moreno coll., 1 ma, 4 fe (MCZ 3125). Región de Araucanía, *Provincia de Cautín*, 27.I.1985, N.I. Platnick & O.F. Francke coll., 1 ma (AMNH); Same, 6.XII.1992, T. Cekalovic & S. Gonzalez coll., 1 ma (AMNH); Same, Fundo de las Selvas, 16–20.II.1981, L.E. Pena coll., 3 ma, 1 fe (AMNH); Same, Fundo de las Selvas, 16–20.II.1981; L.E. Pena coll., 3 ma, 1 fe (AMNH); Same, Villarrica (-39.03, -72.121), 1–30.I.1965, L. Peña coll., 1 ma (MCZ 38265); Same, (-39.03, -72.121), 1–30.I.1965, L. Peña coll., 5 ma (MCZ 31263); Same, 1 ma (MCZ 31264); Same, 6 fe, 4 ma (MCZ 31232); Same, 1 ma, 3 fe (MCZ 31270); Same, Flor del Lago Ranch (-39.2044664427, -72.1279383487), 11.XII.2003, I. Avila & D. Silva coll., 1 ma, 2 fe (CAS 9055035); Same, 2 ma (CAS 9055009); Same, Pucón, 12.I.1951, E. Ross & A. Michelbacher coll., 3 ma, 7 fe (CAS 9055052); Same, Angol, Sierra Nahuelbuta, 13.I.1951, E. Ross & A. Michelbacher coll., 1 ma (CAS, 9055400); Same, Angol (-37.829000, -73.007278), 25–27.XII.2016, A. Anker & P.H. Martins coll., 1 fe (UFMG 22643); Same, 2 fe (UFMG 22644); same, 1 ma (UFMG 22645); Same, 1 ma, 1 fe (UFMG 22646); same, 2 fe (UFMG 22647); Same, 1 ma (UFMG 22649); Same, Temuco, 8.I.1951, E. Ross & A. Michelbacher coll., 1 ma (CAS 9052224); Same, Cerro Ñielol (-38.726389, -72.590833), 26.I.2010, R. Pinto-da-Rocha, F. Cádiz L. & D. Cádiz L. coll., 5 ma, 5 fe (MZSP 36836); Same, *Provincia de Malleco*, Caracatín, VI.1975, collector unknown, 1 ma, 1 fe (AMNH); Same, Purén, Monumento Natural Contulmo, (-38.01256, -73.18517), 12.XI.2014, G. Giribet, G. Hormiga & A. Pérez-González coll., 1 ma, 1 fe (MCZ 138066); Same, Monumento Nacional Contulmo (-38.012778, -73.187500), 12.XII.2010, F. Marques, F. Cádiz & F. Carbayo coll., 9 ma, 10 fe, 3 juvs. (MZSP 36967). Región de Los Lagos, *Provincia de Llanquihue*, 13.II.1994, T. Cekalovic coll., 6 ma, 9 fe (AMNH). Región de Los Ríos, *Provincia de Valdivia*, date or collector unknown, 2 ma, 4 fe (SMF 783); Same, 2 ma, 3 fe (SMF 778); Same, 28.II.1993, T. Cekalovic coll., 2 ma (AMNH); Same, (-39.81389, -73.24583), 15–20.XI.1978, E. Krahmer coll., 3 ma, 4 fe (MCZ 31274); Same, Panguipulli, Puerto Fuy, 24.II.1978, collector unknown, 1 ma (AMNH); Same, Valdivia, Isla Teja (-39.81389, -73.24583), 6.III.1965, H. Levi coll., 1 ma (MCZ 31231).

##### Diagnosis.

*Sadocus asperatus* resembles *S. ingens*, *S. polyacanthus*, and *S. dilatatus* by the bifid prodorsal apical apophysis on coxa IV. *S. asperatus* can be distinguished from the latter species by the combination of the following characters: lateral margin of dorsal scutum covered by granules; trochanter IV with a blunt retro-dorsal apical apophysis being half of the podomere length, and a rhombus retro-ventral apical tubercle; femur IV curved (in dorsal view), with a retro-lateral row of spiniform apophysis (the middle one longest).

##### Redescription.

**Male** (CAS 9055035). Measurements. Dorsal scutum maximum length 6.3; dorsal scutum maximum width 7.2; prosoma maximum length 2.5; prosoma maximum width 3.2; leg femora I 3.2; II 6.5; III 5.5; IV 7.2. ***Dorsum*** (Fig. 3A). Dorsal scutum type gamma triangular. Carapace with granules sparsely distributed. Scutal areas I–IV with eight, four, two and four granules, respectively; scutal area III with one pair of paramedian spiniform tubercles; scutal area IV incompletely divided. Lateral margin of dorsal scutum mostly covered in granules (from posterior half of carapace to posterior margin of dorsal scutum). Posterior margin of dorsal scutum and free tergite I each with a pair of paramedian tubercles. ***Chelicerae*.** Segment I with basal tubercle, bulla with small setae, each finger with five teeth. ***Pedipalps*.** Coxa dorsal face smooth, ventral face with two apical tubercles. Trochanter dorsal and ventral faces smooth. Femur ventral face granulate. Patella with sparsely distributed setae. Tibial setation: prolateral IiiiIi/IiiiIi; retro-lateral iIiIi/IiIi. Tarsal setation: prolateral and retro-lateral IiIi/IiIi. ***Legs*** (Fig. 3B–E). Coxa IV with one long, oblique, bifid prodorsal apical apophysis and one retro-ventral apical spine. Trochanters I and II each with one pair of prodorsal spiniform tubercles. Trochanter III with one medio-ventral tubercle and three retro-lateral ones. Trochanter IV prodorsal and proventral faces with few granules, the prodorsal apical apophysis long (ca. half the podomere length); retro-lateral face with one basal, one central, and one apical tubercles; one retro-dorsal apical spiniform apophysis (ca. ¼ podomere length); ventral face with setiferous tubercles sparsely distributed. Femur III with one retro-basal tubercle. Femur IV sigmoid, with dorsal row of apophysis on the basal half abruptly decreasing in size apically, becoming granules; prolateral row with central–subapical tubercles; retro-lateral row of granules with spiniform apophysis (basal most and central one longer than the others, apical most oblique, curved ventrad); ventral face with two short retro-lateral sub-apical spiniform apophyses and one proapical spiniform apophysis. Patella IV ventral face mostly smooth, with one probasal, one proapical and one retro-apical large, spiniform tubercles. Tibia IV roughly with two ventral rows of granules increasing in size from central to apical becoming spines. Tarsal counts: 6, 9, 7, 8. ***Penis*** (Fig. 11A, B). Ventral plate of penis with moderate cleft on anterior margin, three pairs of MS A, one pair of MS B, four or five pairs of MS C and one pair of MS D, without MS E.

***Coloration*.** Immersed in ethanol: carapace, trochanter–patella IV brown, tibia light brown, legs I–III, pedipalps, and chelicerae yellowish-brown. Live specimens (Fig. 9A–C, E): carapace, coxa, and trochanter black, light gray spot (inverted T shape) from ocularium to scutal area II; femora I–III orange; patellae–tibiae I–IV brown.

***Variations*** (n = 56). Scutal area I–IV tubercles (5 minimum, 16 maximum per site), Measurements. Dorsal scutum maximum length 6.0–8.5; dorsal scutum maximum width 6.7–11.6; prosoma maximum length 2.5–3.5; prosoma maximum width 3.2–4.8; leg femora: I 3.2–5.0; II 6.5–10.0; III 5.5–8.5; IV 7.2–11.0.

*Female* (CAS 9055035). Measurements. Dorsal scutum maximum length 6.3; dorsal scutum maximum width 8.1; prosoma maximum length 2.6; prosoma maximum width 3.7; leg femora: I 3.7; II 7; III 6.1; IV 7.2.

##### Redescription.

***Dorsum*** (Fig. 8A, B). Scutal areas I, II, and IV with three, five, and two granules, respectively. ***Legs*.** Coxa IV with one prodorsal apical apophysis and one retro-ventral apical spine shorter than on the male, trochanter IV with retro-lateral row of tubercles, the apical one longest, femur IV with pro- and retro-ventral rows of tubercles, patella–metatarsi IV unarmed. Tarsal counts: 6, 9, 7, 8. ***Ovipositor*** (Fig. 12A–C). Two main groups of lobes delimited by a constriction, ovipositors peripheral setae inserted into sockets that are a mixture of dorsal and ventral sockets, the dorsal lobe with five setae and the ventral one with six; each main group of lobes divided by a fissure.

***Variations*** (n = 66). Tubercle variation in the scutal areas: I 2–7; II 1–8; III 2–8; IV 3–5. Free tergites I–III each with one pair of blunt or pointed spiniform tubercles. Measurements. Dorsal scutum maximum length 5.9–8.2; dorsal scutum maximum width 8.0–9.6; prosoma maximum length 2.4–3.5; prosoma maximum width 3.7–4.5; leg femora: I 3.5–4.5; II 6.7–8.7; III 5.7–7.0; IV 7.0–8.9.

##### Type locality.

Of *Gonyleptes asperatus* and *Gonyleptes subsimilis*: CHILE. Of *Discocyrtus calcitrosus*: CHILE, Región de Los Rios, *Provincia de Valdivia*, Corral.

##### Geographical distribution

**(Fig. 2).** Chile, Región de Los Ríos, *Valdivia*, Corral; Región Metropolitana de Santiago, *Santiago*; Región de Valparaíso; Región de Biobío, *Provincia de Concepción*, *Provincia de Arauco*, *Provincia de Nuble*; Región de Araucanía, *Provincia de Cautin*, *Provincia de Malleco*; Región de Los Lagos, *Provincia de Llanquihue*.

##### Taxonomic notes.

After examining the original description and the drawing of *Gonyleptes bicornis*, we concluded that it is of a male of *S. asperatus*. In the original description, the spines on the free tergite, the two apical apophyses on the trochanter IV and uneven spines in the inner part of the “leg” (referring to the femur IV) are mentioned. Those characters lead us to conclude that it is *S. asperatus*.

#### Sadocus dilatatus

Taxon classificationAnimaliaOpilionesGonyleptidae

Roewer, 1913

93FAED9C-954D-518C-BB11-51CCCE44271D

**Figure 4.**
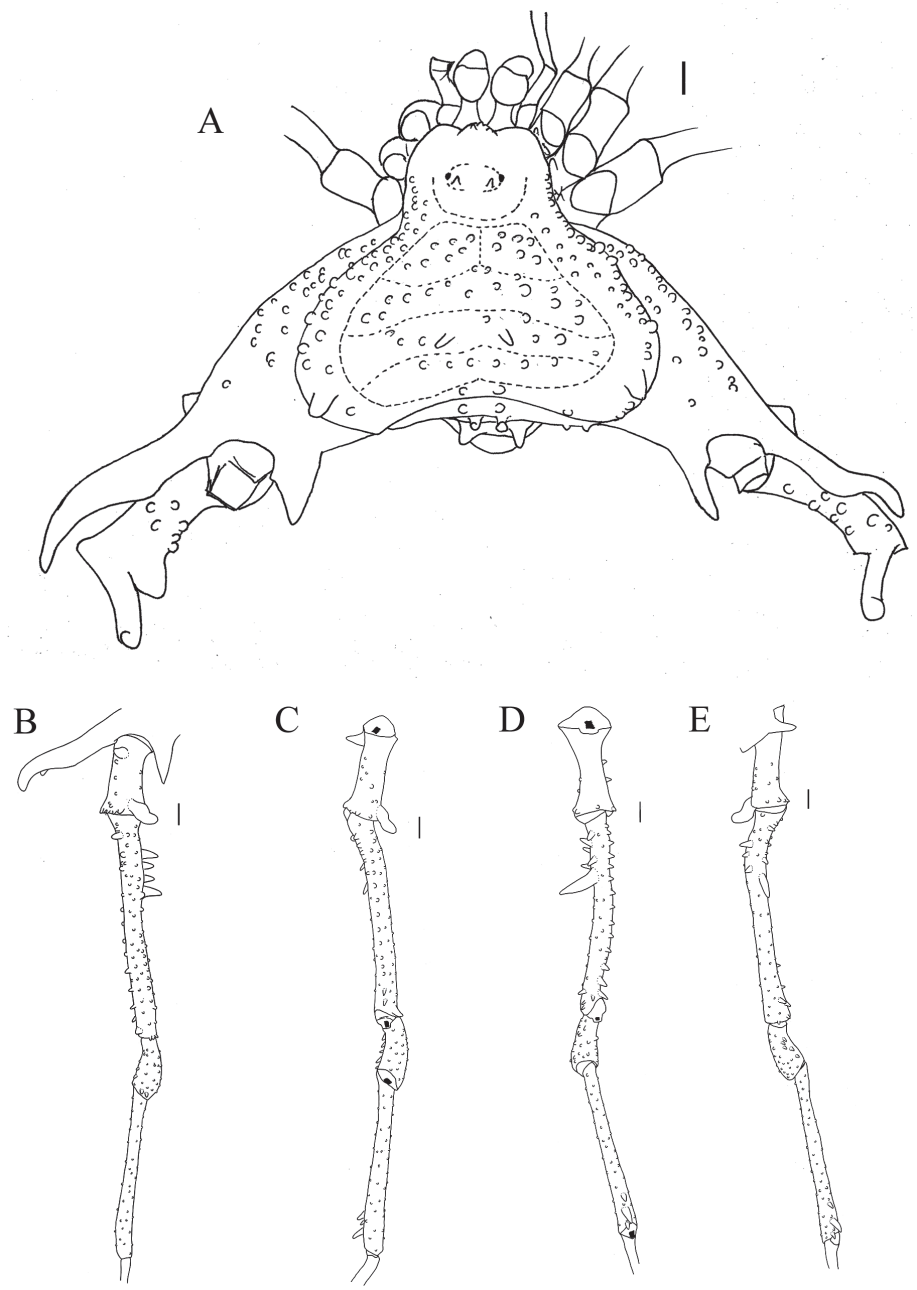
*Sadocus dilatatus* Roewer. Male holotype (SMF 886) **A** habitus, dorsal view **B–E** male left trochanter–tibia IV **B** dorsal view **C** prolateral view **D** ventral view **E** retro-lateral view. Scale bars: 1 mm.

Figures 2 , 4A–E , 8C, D , 9D , 11G, H

Sadocus dilatatus Roewer, 1913: 249, fig 102 [desc]; 1923: 493–494, fig 620 [rdesc]; Canals 1936: 70 [cat]; Soares and Soares 1949: 211 [cat]; Cekalovic 1968: 7 [cat]; Acosta 1996: 223 [cat]; Kury 2003: 191 [cat]; Kury et al. 2020b [cat] (SMF RI, 886, ma holotype – examined).
Lycomedicus dilatatus : H. Soares, 1968: 264 [rdesc]; Cekalovic 1985: 18 [cat].

##### Material examined.

Chile, Región de Biobío, *Provincia Concepción*, date or, collector unknown, 1 ma (SMF 886 – Holotype); Same, 1.XI.1964, T. Cekalovic coll., 2 ma, 1 fe (MZSP 7875); Same, Quebrada Pinares, 4.XI.1964, T. Cekalovic coll., 1 ma (MZSP 7876); Same, Reserva Nacional Nonguén (-36.878430, -72.994350), G. Giribet, G. Hormiga & A. Pérez-González coll., 11.XI.2014, 1 ma, 2 fe (MCZ 140078).

##### Diagnosis.

*Sadocus dilatatus* resembles *S. polyacanthus* by the lesser-armed femur IV (compared to other species) and by the posterior large tubercle on the lateral margin of dorsal scutum. *Sadocus dilatatus* can be distinguished from the other species of the genus by the single retro-ventral central apophysis on femur IV and the very long prodorsal apical apophysis on coxa IV (ca. ⅔ of the scutum width).

##### Redescription.

**Male** (SMF 886). ***Measurements*.** Dorsal scutum maximum length 7.5; dorsal scutum maximum width 10.4; prosoma maximum length 3.2; prosoma maximum width 4.2; leg femora: I 6.0; II 13.0; III 10.3; IV 11.0. ***Dorsum*** (Fig. 4A). Dorsal scutum type gamma triangular. Anterior margin of dorsal scutum with median frontal hump bearing six tubercles and three granules on each side. Carapace with granules sparsely distributed. Scutal areas I–IV with 13, 16, eight and six granules, respectively; scutal area III with one pair of paramedian spines; scutal area IV completely divided (from area III). Lateral margin of dorsal scutum mostly covered by granules (from the posterior half of carapace to posterior margin of dorsal scutum), with one large tubercle near scutal area IV. Posterior margin of dorsal scutum and free tergite I each with few granules on the corners. ***Chelicerae*.** Segment I with one seta on mesal side of the bulla, each finger with five or six teeth. ***Pedipalps*.** Coxa mostly smooth, with one ventro-central tubercle. Trochanter dorsal face smooth, with one retro-ventral apical tubercle and one retro-apical spine. Femur dorsal face smooth, ventral row of few granules and one retro-ventral spine. Patella smooth. Tibial setation: prolateral Iiii/IiiIi; retro-lateral IiIi/IiIi. Tarsal setation: prolateral IiIii/IiIi; retro-lateral iiIiIii/iIiIii. ***Legs*** (Fig. 4B–E). Coxa IV with one long, oblique, bifid prodorsal apical apophysis and one retro-ventral apical spine. Trochanters I–III granulate; trochanter III with one retro-apical spine. Trochanter IV with sparse granules, the prodorsal apical apophysis long, (ca. ⅓ of podomere length), pointing prolaterad. Femur III with proventral and retro-ventral rows of granules increasing in size apically, becoming tubercles. Femur IV with three blunt dorsal spines on basal ⅓; proventral row of pointed granules increasing in size apically becoming spines; retro-lateral row with two spines and one central apophysis and one proventral basal spine. Patella IV with ventral row of tubercles. Tibia IV with three retro-ventral apical spines (apical one the largest). Tarsal counts: 6, 11, 7, 10. ***Penis*** (Fig. 11G, H). Ventral plate of penis with attenuated cleft on anterior margin; three pairs of MS A, one pair of MS B, four pairs of MS C, one pair of MS D, and two pairs of ventral MS E.

***Coloration.*** Immersed in ethanol: carapace and leg IV dark brown; legs I–III, pedipalps and chelicerae light brown. Specimen color badly preserved. Live specimens (Fig. 9D): carapace, coxa, and trochanter black, dry-mark on carapace; femora I–III, patellae–tibiae I–IV brown; border of dorsal scutum and free tergite green; free tergite yellowish.

***Variations*** (n = 4). Scutal areas I–IV with 10–13, 14–16, 8–10, 5–6 granules, respectively. In smaller males, the lateral margin of dorsal scutum may bear cluster of tubercles instead of a large tubercle. Measurements. Dorsal scutum maximum length 7.0–8.1; dorsal scutum maximum width 8.5–11.5; prosoma maximum length 3.0–3.4; prosoma maximum width 4.1– 4.4; leg femora: I 5.5–6.5; II 11.4–14.5; III 9.0–11.5; IV 9.7–12.0.

**Female** (MZSP – 8022). ***Measurements*.** Dorsal scutum maximum length 7.5; dorsal scutum maximum width 8.9; prosoma maximum length 3.4; prosoma maximum width 4.5; leg femora: I 5.0; II 11.4; III 9.0; IV 11.0. ***Dorsum*** (Fig. 8C, D). Lateral margin of dorsal scutum with five slightly large granules. Scutal areas I–IV with ten, twelve, eleven, and seven granules, respectively. ***Legs*.** Coxa IV with moderate prodorsal spiniform apophysis (as long as the podomere width, but shorter than male) and one retro-ventral apical spine shorter than on the male. Trochanter IV with a retro-apical apophysis. Femur III with proventral and retro-ventral row of granules increasing in size apically, becoming tubercles; femur IV with prolateral row of pointed granules on distal half and a retro-lateral row of pointed granules on basal half. Tarsal counts: 6, 11, 7, 9.

##### Type locality.

Chile, Región de Biobío, *Provincia Concepción*.

##### Geographical distribution

**(Fig. 2).** Chile. Región de Biobío, *Provincia Concepción*, Quebrada Pinares.

##### Note.

The allotype MZSP 7874 was not used for the variation or in the distribution maps because it is a female, which lacks the diagnostic characters of the species (solely based on male characters).

##### Erratum.

H. Soares (1968) incorrectly cited the collection number 7676 for the vial labelled: CHILE, Región de Biobío, *Provincia Concepción*, Quebrada Pinares, 4.XI.1964, T. Cekalovic coll., 1 ma. It is in fact 7876. The vial with number 7875 mentions 2 ma, when in fact, it includes 2 ma and 1 fe, and vial 7874 mentions only 1 ma, when it is in fact 1 fe.

#### Sadocus funestus

Taxon classificationAnimaliaOpilionesGonyleptidae

(Butler, 1874)

102459D9-B540-512A-B435-7C502DFB07D1

**Figure 5.**
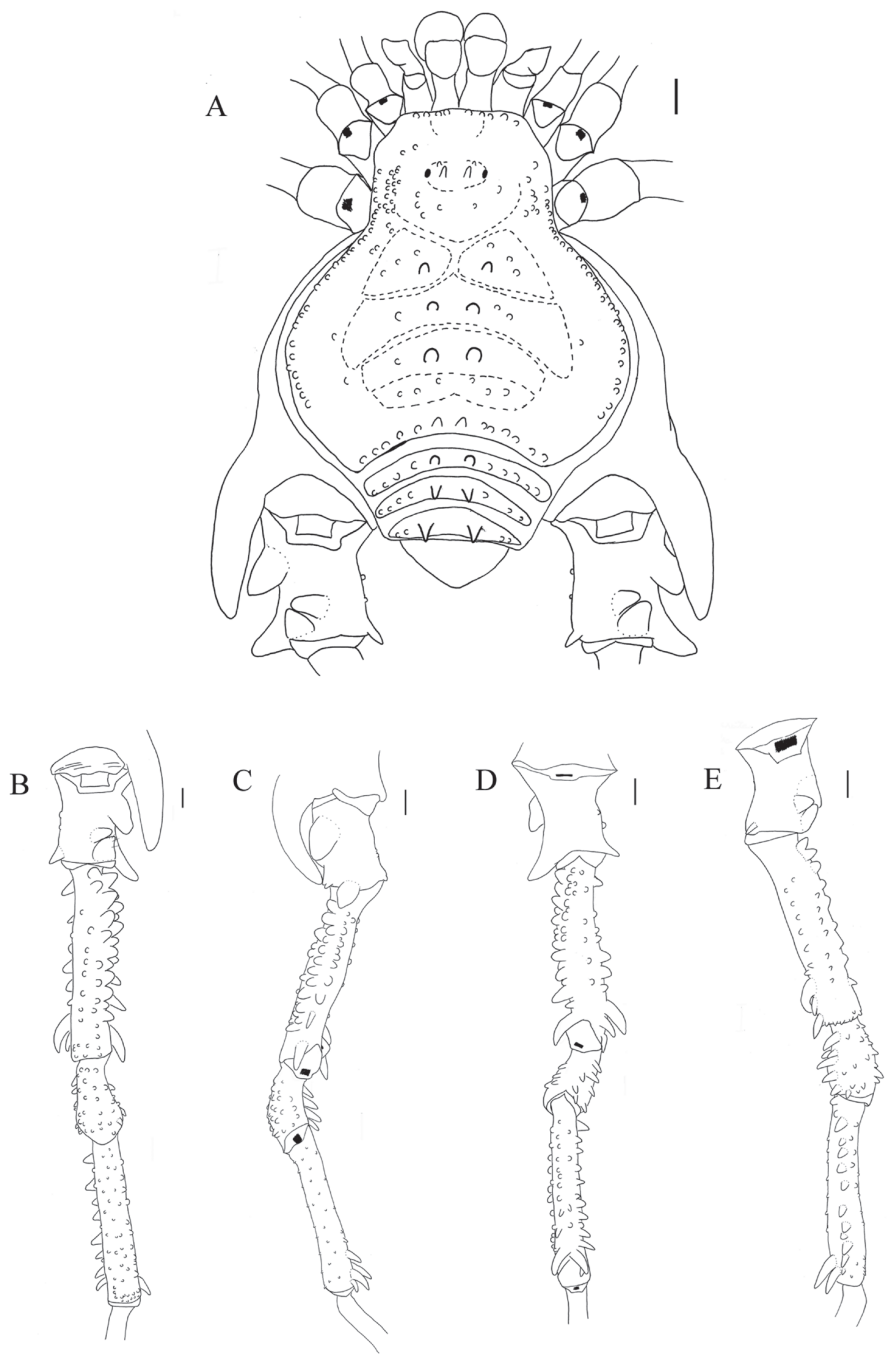
*Sadocus funestus* Butler. Male (CAS 9026265) **A** habitus, dorsal view **B–E** male right trochanter–tibia IV **B** dorsal view **C** prolateral view **D** ventral view **E** retro-lateral view. Scale bar: 1 mm.

Figures 2 , 5A–E , 8E, F , 9F , 11I–J , 12D–F

Gonyleptes funestis Butler, 1874: 153, figs 5, 5a [desc]; Loman 1899: 6, fig 3 [cit]; Sørensen 1902: 20 [cit]. (Type material NHM, ma holotype, examined by detailed photographs).
Discocyrtus funestus : Loman, 1899: 6, fig 3 [syst]; Sørensen 1902: 20 [syst].
Lycomedes funestus : Sørensen, 1902: 20 [syst]; Roewer 1913: 127, 130, fig 58 [rdesc].
Lycomedicus funestus : Roewer, 1923: 443, fig 557 [rdesc]; 1929: 214 [cat]; Canals 1936: 69 [cat]; Soares and Soares 1954: 271 [cat]; Muñoz-Cuevas 1973: 232, fig 4 [cit]; Cekalovic 1968: 7 [cat]; 1985: 18 [cat]; Cokendolpher 1993: 136 [eco].
Sadocus funestis : Kury, 2003: 191 [cat]; Pérez-González et al. 2020: 3 [cit].
Sadocus funestus : Kury et al., 2020b [cat].

##### Material examined.

Chile, Región de Los Ríos, *Provincia de Valdivia*, Corral, date or collector unknown, 1 ma (SMF 766); Same, Valdivia, date or collector unknown, 1 ma (SMF 276); Same, Curiñanco, 12.I.2006, Elizabeth Arias et al. coll., 1 ma, 2 fe, 3 juvs. (AMNH); Same, Oncol Park (-39.70025, -73.0), 12.I.2006, Elizabeth Arias et al. coll., 2 ma, 3 fe (AMNH); Same, 7.II.2004, T. Cekalovic coll., 2 ma (CAS 9026265); Same, 1 ma (AMNH); Same, 10.I.2006, Elizabeth Arias et al. coll., 1 ma, 2 fe (AMNH); Same, 7.II.2004, T. Cekalovic coll., 4 ma, 1 fe (AMNH); Same, (-39.712333, -73.307278), 01.I.2017, A. Anker & P.H. Martins coll., 1 ma (UFMG 22650); Same, 1 ma (UFMG 22651); Same, 2 ma (UFMG 22652); Same, 2 ma (UFMG 22653); Same, on the way to Oncol Park, 15.II.2004, T. Cekalovic coll., 1 ma (AMNH); Same, Chiguayco, I.1980, collector unknown, 1 ma (AMNH); Same, Las Lajas, Las Tablas, 9.I.1989, L.S. Kimsey coll., 1 ma, 3 fe (MCZ 31262); Región da Araucanía, Villarrica (-39.03 -72.121), 1–30.I.1965, L. Peña coll., 1 ma (MCZ 38272); ECUADOR, Canelos [doubtful record], date or collector unknown, 1 ma (SMF 777).

##### Diagnosis.

*Sadocus funestus* can be distinguished from other species of the genus by the following characters: uniramous prodorsal apical apophysis on coxa IV; leg IV straight; and trochanter IV with four apophyses.

##### Redescription.

**Male** (CAS 9026265). ***Measurements*.** Dorsal scutum maximum length 9.3; dorsal scutum maximum width 10.0; prosoma maximum length 3.9; prosoma maximum width 5.0; leg femora: I 4.5; II 8.3; III 7.0; IV 8.0. ***Dorsum*** (Fig. 5A). Dorsal scutum type gamma pyriform. Dorsal scutum anterior margin with nine granules, lateral margins with a row of granules and cluster of granules near scutal groove I. Ocularium with one pair of granules on anterior face. Scutal areas I–IV with six (three on each side), three, one, and six granules, respectively; scutal areas I–III each with one pair of paramedian tubercles. Scutal area IV completely divided. Lateral margin of dorsal scutum with row of granules between ozopore area and anterior part of scutal area IV. Posterior margin of dorsal scutum and free tergites I–III each with a row of granules. ***Chelicerae*.** Segment I with setae on bulla; fixed finger with four teeth, movable finger with three teeth. ***Pedipalps*.** Coxa with one ventro-apical spine. Trochanter dorsal face smooth, with one pair of geminated ventro-apical setiferous tubercles. Femur with a row of ventro-basal granules. Patella smooth. Tibial setation: prolateral IiIi/IiiI; retro-lateral iIiIi/iIiIi. Tarsal setation: prolateral IiIii/IiIi; retro-lateral iiIIIiii/iiIiiIii. ***Legs*** (Fig. 5B–E). Coxa IV with one long, oblique, uniramous prodorsal apical apophysis, without retro-ventral apical spine. Trochanters I and III granulate; trochanter III with one retro-apical spine. Trochanter IV proapical spiniform apophysis with large base (ca. ⅓ of podomere length); additionally with two retro-dorsal apical apophyses with the ridges touching each other; retro-lateral face with two central granules and one apical spine. Femur III dorsal face granulate; ventral face with two rows of granules increasing in size apically. Femur IV with prodorsal row of large tubercles decreasing in size apically; prolateral row of tubercles; one retro-ventral row of granules; proventral row of granules increasing in size apically; one proventral apical spiniform apophysis, one retro-sub-apical spiniform apophysis, and two or three retro-ventral apical apophyses. Patella IV with five retro-ventral spines. Tibia III with ventral row of tubercles. Femur IV with retro-lateral row of spines and four ventro-apical spines. Tarsal counts: 6, 12, 7, 8. ***Penis*** (Fig. 11I, J). Ventral plate of penis with deeper (than moderate) cleft on anterior margin, three pairs of MS A, four pairs of MS C, one pair of MS D, without MS B or E.

***Coloration*.** Immersed in ethanol: carapace, trochanter, and leg IV brown; legs I–III, pedipalps fading from brown to yellowish. Tubercles and spines of dorsal scutum and free tergites yellowish. Live specimens (Fig. 9F): carapace, coxa, and trochanter black, dry-mark on dorsal scutum; femora I–III, patellae–tibiae I–IV brown; free tergite green.

***Variations*** (n = 21). Granules between lateral margin of carapace and ocularium varying from none to 4–19. Color of granules on carapace range from brown to yellowish. Measurements. Dorsal scutum maximum length 8.3–10.0; dorsal scutum maximum width 8.6–10.5; prosoma maximum length 3.4–4.0; prosoma maximum width 4.7–5.5; leg femora: I 4.5–5.0; II 8.3–9.4; III 7.0–7.9; IV 7.5–8.5.

**Female** (AMNH CHILE, Región de Los Rios, *Provincia Valdivia*, Parque Oncol, 10.I.2006, Elizabeth Arias et al. coll., 1 ma, 2 fe). ***Measurements*.** Dorsal scutum maximum length 8.4; dorsal scutum maximum width 10.0; prosoma maximum length 3.4; prosoma maximum width 5.0; leg femora: I 5.0; II 9.5; III 7.8; IV 9.7. ***Dorsum*** (Fig. 8E, F). Lateral margin of dorsal scutum with two rows of tubercles. ***Legs*.** Coxa IV with short prodorsal apical apophysis, seen in a ventral view. Trochanter IV prolateral and dorsal faces unarmed, with one retro-lateral spiniform apophysis. Femora III–IV with a prolateral and a retro-lateral rows of tubercles, increasing in size apically. Tarsal counts: 5, 11, 7, 8.

***Variations*** (n = 8). Dorsal scutum with 10–24 granules, the armature on the free tergites varying from one paramedian pair of blunt to pointed tubercles. Measurements. Dorsal scutum maximum length 5.5–8.3; dorsal scutum maximum width 8.7–10.0; prosoma maximum length 3.4–3.5; prosoma maximum width 4.5–5.0; leg femora: I 4.8–5.0; II 9.0–9.5; III 7.5–7.8; IV 9.3–9.7.

##### Geographical distribution

**(Fig. 2).** Chile, Región de Los Ríos, Provincia de Valdivia, Curiñanco & Chiguayco. The record for Ecuador is doubtful (Kury 2003).

#### Sadocus ingens

Taxon classificationAnimaliaOpilionesGonyleptidae

(Mello-Leitão, 1937)

9AB1D486-3FDB-5469-94F2-511D067E03A1

**Figure 6.**
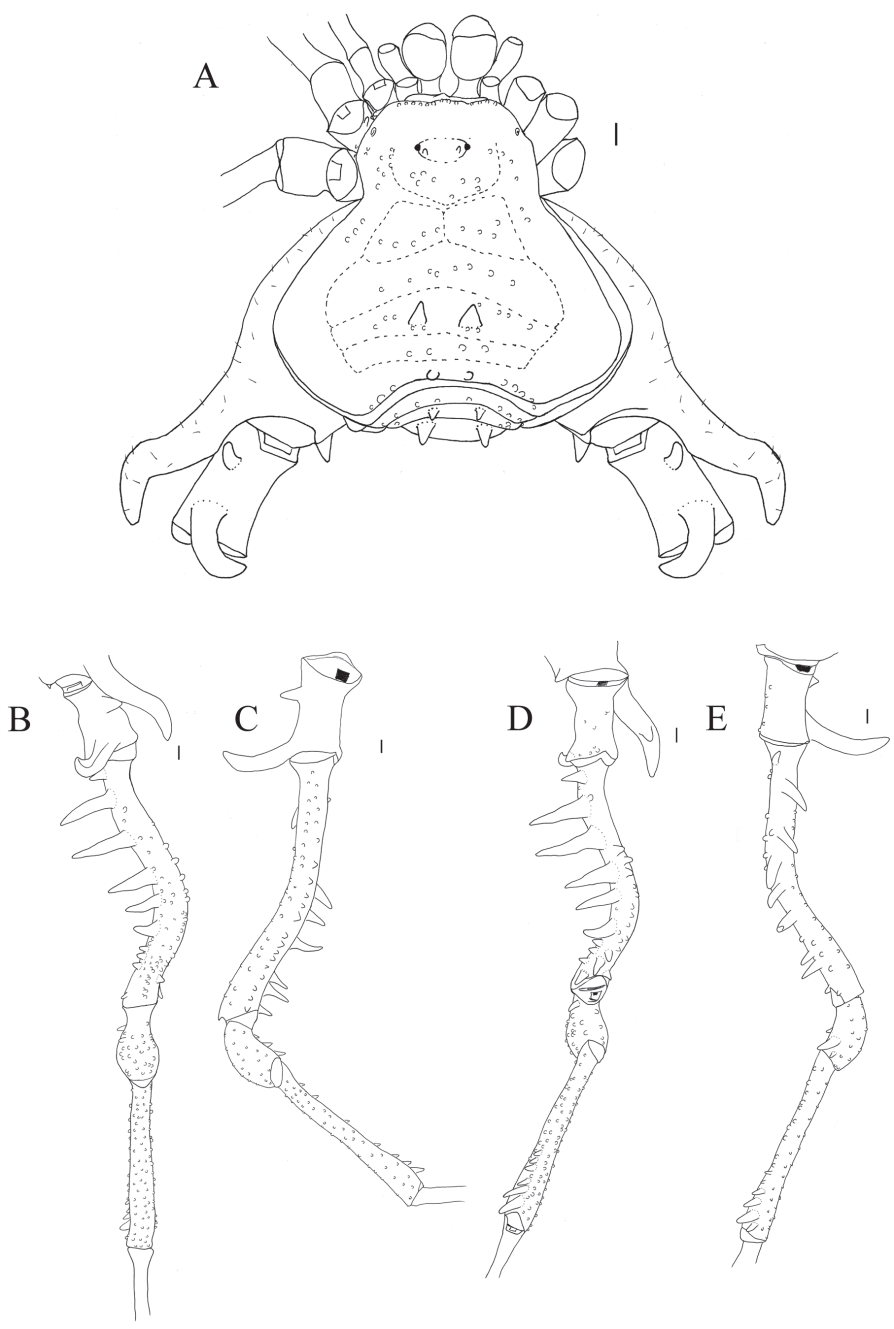
*Sadocus ingens* (Mello-Leitão). Male (MZSP 36965) **A** habitus, dorsal view **B–E** male right trochanter–tibia IV **B** dorsal view **C** prolateral view **D** ventral view **E** retro-lateral view. Scale bars: 1 mm.

Figures 2 , 6A–E , 8G, H , 10A, B , 11C, D , 12G–I

Carampangue ingens Mello-Leitão, 1937: 152–153, figs 14–15 [desc]; B. Soares 1945: 370 [cat]; Mello-Leitão 1949: 17 [syst]; Soares and Soares 1954: 242 [cat]; Cekalovic 1968: 7 [cat]; 1985: 17 [cat] (type MNRJ 5263, 1 ma, 1 fe, syntype, previously preserved in dry collection, examined).
Sadocus ingens : Kury, 2003; 191 [cat]; Kury et al. 2020b [cat].
Jighas vastus Roewer, 1943: 28, pl. 3, fig 22 [desc]; Mello-Leitão 1949: 17 [syst]; Acosta 1996: 217 [cat]. (type SMF RII 1380/73, 2 ma, 2 fe, syntypes, examined). Synonymy established by Mello-Leitão 1949.

##### Material examined.

Chile, Región de Araucanía, *Provincia de Malleco*, Contulmo Natural Monument (-38.012778, -73.187500), 12.XII.2010, F. Marques, F. Cadiz & F. Carbayo coll., 1 ma, 1 fe (MZSP 36965); Same, Purén, Salto el Rayen (-38.013528, -73.162639), 04.I.2017, A. Anker & P.H. Martins coll., 1 ma (UFMG 22654); Same, 1 ma (UFMG 22655); Same, *Provincia de Cautín*, Temuco, 1943, collector unknown, 2 ma, 2 fe (SMF 1380/73); Same, Región de Biobío, *Provincia de Arauco*, Carampangue, 1937, W. Feed coll., 1 ma 1 fe (MNRJ 5263 – Syntypes).

##### Diagnosis.

*Sadocus ingens* can be distinguished from the other species of the genus by being the largest among them (and quite large among gonyleptid harvestmen); by the prodorsal apical apophysis on trochanter IV of the same length as the podomere (in other *Sadocus* species, that apophysis length is up to ½ the podomere length); lateral margin of dorsal scutum smooth posterior to scutal area II.

##### Redescription.

**Male** (MZSP 36965). ***Measurements*.** Dorsal scutum maximum length 12.0; dorsal scutum maximum width 15.5; prosoma maximum length 4.5; prosoma maximum width 7.2; leg femora: I 8.7; II 17.4; III 11.7; IV 19.4. ***Dorsum*** (Fig. 6A). Dorsal scutum type gamma pyriform. Anterior margin of dorsal scutum with 13 granules, lateral margin of dorsal scutum with three to nine granules on carapace, seven granules behind ocularium. Scutal areas I–IV with four or five, nine, six, and four granules, respectively; scutal area III with one paramedian pair of spines. Scutal area IV completely divided by fading scutal groove IV. Lateral margin of dorsal scutum and free tergites I and II with three or four, eight and two granules, respectively. Free tergite III smooth. ***Chelicerae*.** Segment I with one probasal spine on bulla, one retro- and one prolateral pair of filiform spines; segment II, fixed finger with four teeth, movable finger with three teeth. ***Pedipalps*.** Coxa smooth and barely visible. Trochanter ventral face with one retro-apical tubercle and one prolateral spine. Femur dorsal face granulate, one ventro-basal tubercle, retro-ventral row of granules and a ventral row of tubercles. Patella smooth. Tibial setation: prolateral IiIi/IiiIi; retro-lateral iIiIi. Tarsal setation: prolateral iIiIi; retro-lateral iIiIii/iiIiIii. ***Legs*** (Fig. 6B–E). Coxa IV covered by setae, with one robust, long, bifid prodorsal apical apophysis and one short retro-ventral spiniform apophysis. Trochanters I–III granulate; trochanters I and III dorsal face smooth. Trochanter IV with few granules on ventral central and apical areas; one retro-lateral tubercle; the prodorsal apical apophysis long, curved, as long as the podomere length. Femur IV with retro-lateral row of spines (five prominent) decreasing in size apically, becoming blunt tubercles; retro-ventral row of granules with one basal pointed tubercle, few tubercles on the middle ⅓ and two spines on apical area; ventro-apical face with one retro-lateral spine and one pointed, prolateral tubercle. Patella IV with three or four retro-ventral spines. Tibia IV dorsal face granulate and with retro-ventral row of granules increasing in size apically, becoming spines on distal half. Tarsal counts: 8, 16, 8, 8. ***Penis*** (Fig. 11C, D). Ventral plate of penis with moderate cleft on anterior margin, two or three pairs of MS A, four or five pairs of MS C and one pair of MS D.

***Coloration*.** Immersed in ethanol: carapace, trochanters, femora, patella IV, and tibia IV dark brown. Scutal areas II and III, free tergites, patellae and tibiae I–III orange. Live specimens (Fig. 10A, B): carapace, scutal areas and legs I–IV black; lateral margin of dorsal scutum dark brown with green pleurites; posterior margin of dorsal scutum and free tergites orange, arthrodial membranes green.

***Variations*** (n = 6) – Free tergites II and III with one paramedian pair of spines which length varies from similar to slightly longer than the tergite length, its apex varying from blunt to pointed; femur IV with granules in between the retro-lateral spines. Measurements. Dorsal scutum maximum length 12.0–13.8; dorsal scutum maximum width 12.7–16.5; prosoma maximum length 4.5–5.4; prosoma maximum width 7.2–7.8; leg femora: I 8.7–10.0; II 16.8–18.5; III 11.7–15.0; IV 16.0–20.0.

**Female** (MZSP 36965). ***Measurements*.** Dorsal scutum maximum length 12.0; dorsal scutum maximum width 11.5; prosoma maximum length 5.0; prosoma maximum width 7.0; leg femora: I 8.4; II 16.2; III 12.0; IV 17.0. ***Dorsum*** (Fig. 8G, H). Scutal areas I–IV with six, six, four, and four granules, respectively. ***Legs*.** Coxa IV with discreet apophysis, not seen in ventral view. Tarsal counts: 8; 15; 8; 9. ***Ovipositor*** (Fig. 12 G–I). Two main groups of lobes delimited by constriction, ovipositors peripheral setae inserted into sockets that are a mixture of dorsal and ventral sockets, left lobe with six setae and right lobe with six. Each main group of lobes divided by a fissure.

***Variations*** (n = 4). Tubercle variation in the areas: I–2–8, II–4–6, III–3–6, IV–2–4. Measurements. Dorsal scutum maximum length 11.0–12.0; dorsal scutum maximum width 10.0–11.7; prosoma maximum length 4.5–5.0; prosoma maximum width 6.4–7.2; leg femora: I 7.1–8.4; II 13.8–16.2; III 11.0–12.0; IV 13.0–17.0.

##### Type locality.

Chile, Región de Araucanía, Provincia de Malleco, Monumento Nacional Contulmo.

##### Geographical distribution

**(Fig. 2).** Chile. Región de Araucanía, Provincia de Cautín; Idem, Provincia de Malleco, Temuco; Región de Biobío, Provincia de Arauco.

#### Sadocus polyacanthus

Taxon classificationAnimaliaOpilionesGonyleptidae

(Gervais, 1847)

AFF958CD-24CC-506E-A99B-418533DD4398

**Figure 7.**
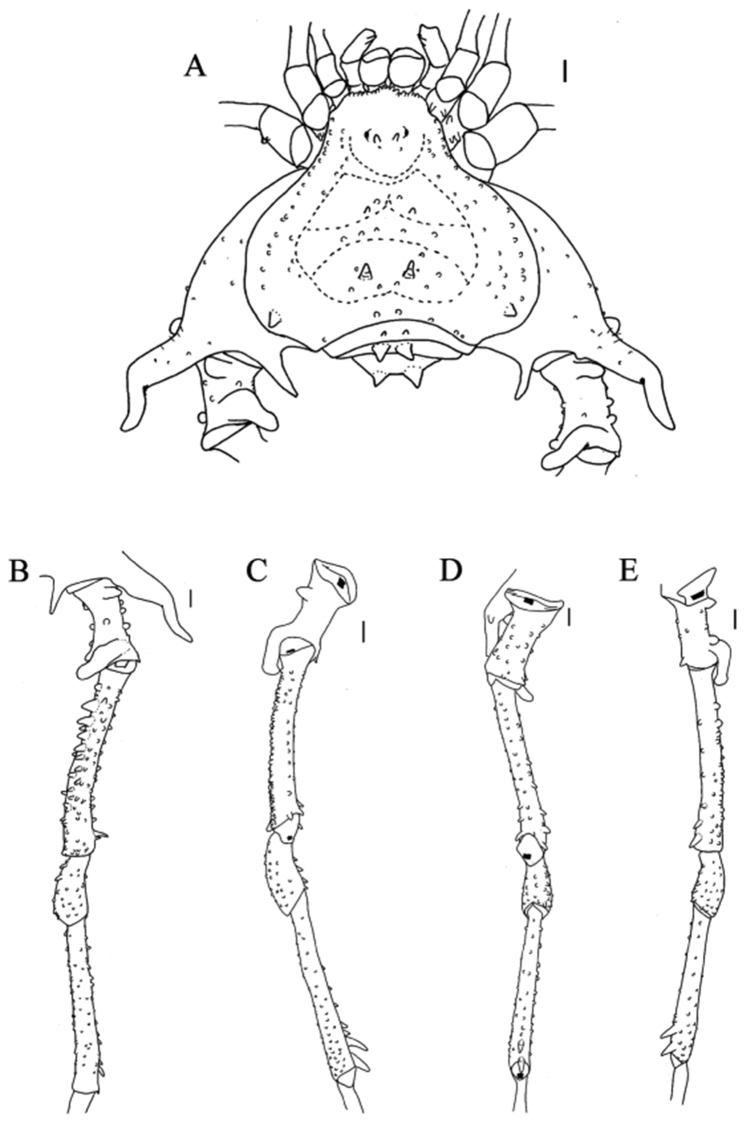
*Sadocus polyacanthus* (Gervais). Male (CAS 9055047) **A** habitus, dorsal view **B–E** male right trochanter–tibia IV **B** dorsal view **C** prolateral view **D** ventral view **E** retro-lateral view. Scale bars: 1 mm.

**Figure 8.**
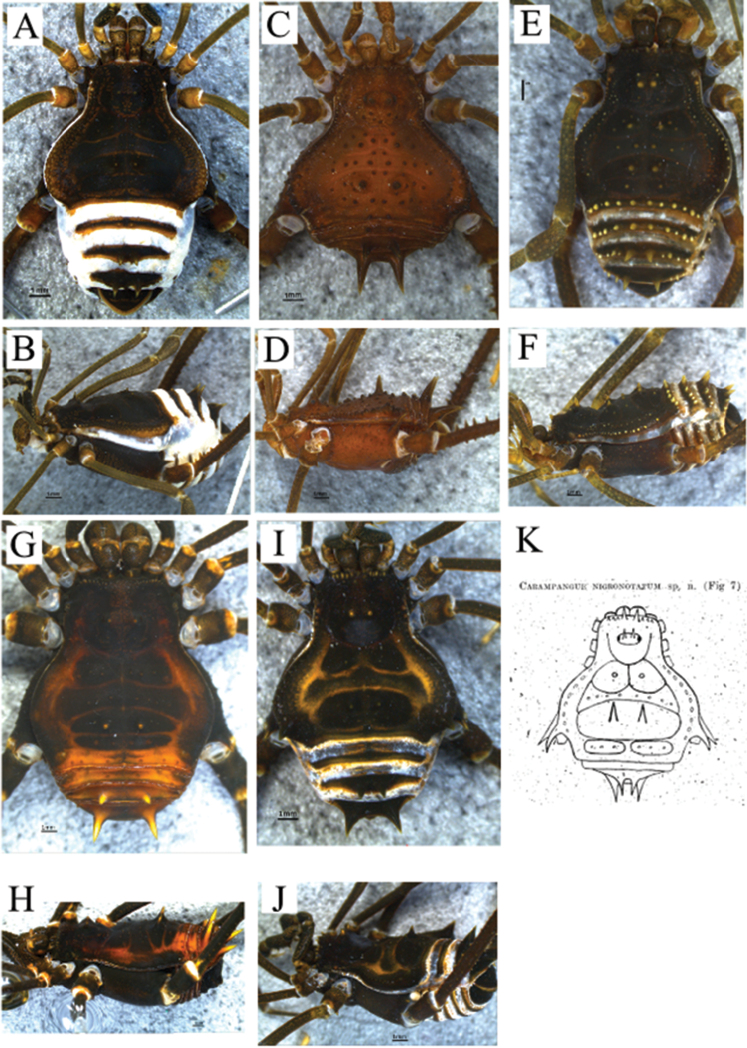
Female specimens of *Sadocus***A, B***S. asperatus* (CAS–9055035) **A** dorsal view **B** lateral view **C, D***S. dilatatus* (MZSP–8022) **C** dorsal view **D** lateral view **E, F***S. funestus* (AMNH) **E** dorsal view **F** lateral view **G, H***S. ingens* (MZSP–36965) **G** dorsal view **H** lateral view **I, J***S. polyacanthus* (AMNH) **I** dorsal view **J** lateral view **K** Drawing of species inquirenda *Carampangue nigronotatum* extracted from Mello-Leitão 1943.

Figures 2 , 7A–E , 8I, J , 10C–F , 11E, F , 12J–L

Gonyleptes polyacanthus Gervais, 1847: 576 [desc]; 1849: 24, pl. 1, figs 7–7c [rdesc]; Butler 1873: 114 [cat]. (Syntypes MNHN, 1 ma, 1 fe examined by detailed photographs). Transferred to Sadocus by Sørensen 1902.
Sadocus polyacanthus : Sørensen, 1902: 14 [syst; rdesc]; Roewer 1913: 245–246, fig 101 [key, rdesc]; 1923: 493, fig 619 [rdesc]; 1930: 381 [cit]; Canals 1934: 6 [cit]; 1936: 70 [cat]; Roewer 1938: 02, 06 [cat]; Mello-Leitão 1939: 625 [cit]; Soares and Soares 1949: 212 [cat]; Ringuelet 1955b: 113 [cit]; 1957: 13, 19 [cit]; 1959: 409, figs XVIII-1, 2 [rdesc]; Cekalovic 1968: 7 [cat]; 1976: 24 [cat]; 1985: 15 [cat]; Acosta and Maury 1998: 569 [cit]; Kury 2003: 191 [cat]; Hara et al. 2012: 38–39 [syst]; Pinto-da-Rocha et al. 2012: 61 [cit]; Pinto-da-Rocha et al. 2014: 18 [syst]; Hara 2016: 106–109 [syst]; Kury et al. 2020b [cat].
Sadocus vitellinosulcatus Sørensen, 1886: 85, pl. 6, fig 7 [desc]. (Type depository unknown, fe). Synonymy with S. polyacanthus established by Sørensen 1902.
Gonyleptes platei Loman, 1899: 5, pl. 5, fig 3–3a [desc]. (Holotype ZMB 7843, 1 ma examined). Synonymy with S. polyacanthus established by Sørensen 1902.
Araucanoleptes exceptionalis Mello-Leitão, 1946: 5, fig 5 [desc]; Soares and Soares 1949: 160 [cat] (Syntypes – URMU 703, 1 ma, 3 fe examined). Transferred to Sadocus by Kury 2003. syn. nov.
Arauconoleptes exceptionalis [spelling mistake]: Cekalovic, 1968: 7 [cat]; 1985: 12 [cat].
Sadocus platei : Moritz, 1971: 207 [cat].
Sadocus conspicillatus Roewer, 1913: 251 – 253, pl. 1, fig 3 [desc]; 1923: 495 [rdesc]; Canals 1936: 70 [cat]; Soares and Soares 1949: 211 [cat]; Cekalovic 1968, 7 [cat]; 1985: 14 [cat]; Pinto-da-Rocha et al. 2012: 61 [cit]; Kury et al. 2020b [cat]. (Syntypes – SMF, 1 ma 1 fe examined). syn. nov.
Sadocus guttatus Sørensen, 1902: 15 [desc]; Roewer 1913: 248 [rdesc]; 1923: 494 [rdesc]; Canals 1936: 70 [cat]; Soares and Soares 1949: 211 [cat]; Roewer 1961: 102 [cit]; Cekalovic 1968, 7 [cat]; 1985: 14 [cat]; Kury 2003: 191 [cat]; Kury et al. 2020b [cat] (Holotype – ZMUC, ma examined). syn. nov.
Sadocus exceptionalis : Kury, 2003: 191 [cat]; Kury et al. 2020b [cat].

##### Material examined.

Chile, Región Metropolitana de Santiago, *Santiago*, date or collector unknown, 4 ma, 2 fe (SMF 1384); Región de Valparaíso, 13.II.89, collector unknown, 1 ma (ZMUC– holotype of *S. guttatus*); Región de Biobío, *Provincia de Arauco*, Parque Nacional Nahuelbuta (-37.805567, -73.03505), 30.XI.2003, I. Avila, S. Ocares & D. Silva coll. (CAS 9055055); Same (-37.806433, -73.036333), date or collector unknown (CAS 9055038); Same (-37.810556, -73.0575), 13.XII.2010, F. Marques, F. Cádiz & F. Carbayo coll., 4 ma, 4 fe (MZSP 36964); Same (-37.8275, -73.009722), 9.XII.2010, F. Marques, F. Cadiz & F. Carbayo coll., 3 ma (MZSP 36838); Same, *Provincia Concepción*, date or collector unknown, 2 ma (SMF 906; holotype of *S. conspicillatus*); Región de la Araucanía, *Provincia de Vilarrica*, Las Ochocientas (-39.1668, -71.98755), 14.XII.2003, E. Arias & D. Silva et al. coll. (CAS 9055047); Same, *Provincia de Malleco*, Lonquimay, 23–24.XII.1976, collector unknown, 1 ma, 1 fe (AMNH); Same (Las Raices), collector unknown, 1 ma (AMNH); Same, Contulmo Natural Monument, 23.I.1985, N.I. Platnick & O.F. Francke coll., 3 ma, 1 fe (AMNH); Same (-40.73715, -72.31062), 12.XI.2014, G. Giribet, G. Hormiga & A. Pérez-González coll., 2 ma, 3 fe (MCZ 138065); Same, Parque Nacional de Tolhuaca (-38.226111, -71.730833), 18.I.2008, R. Pinto-da-Rocha coll., 4 ma, 3 fe, 1 juv (MZSP 29076); Same, 12.XII.2010, F. Marques, F. Cádiz & F. Carbayo coll., 5 ma, 2 fe (MZSP 36966); Curacatín, Salto del Indio no Rio Cautín (-38.46125, -71.741333), 28.XII.2016, A. Anker & P.H. Martins coll., 1 ma, 1 fe (UFMG 22632); Purén, Salto el Rayen (-38.013528, -73.162639), 04.I.2017, A. Anker & P.H. Martins coll., 1 ma (UFMG 22637); Same, 1 fe (UFMG 22640); Same, 1 ma (UFMG 22641); Same, 1 ma (UFMG 22642); Same, *Provincia de Cautin*, Angol, Parque Nacional Nahuelbuta (-37.829000, -73.007278), 25–27.XII.2016, A. Anker & P.H. Martins coll., 1 ma, 1 fe (UFMG 22628); Same, 1 fe (UFMG 22629); Same, 1 ma (UFMG 22630); Same, 1 fe (UFMG 22631); Same, Fundo de las Selvas, 16–20.II.1981, L.E. Peña coll., 3 ma (AMNH); Same, Vulcão Villarrica, 15–29.XII.1982, A. Newton & M. Thayer coll., 1 ma (AMNH); Same, Villarrica (-39.03 -72.121), 16–31.XII, L. Peña coll., 1 ma, 1 fe (MCZ 38270); Same, 1–30.I.1965, 4 ma, 3 fe (MCZ 38271); Same, 5 ma, 4 fe (MCZ 38273); Same, 4 ma (MCZ 38272); Same, 2 ma (MCZ 31240); Same, Lago Conguillio, Parque Nacional Conguillio (-38.647778, -71.610278), 24–25.I.2010, R. Pinto-da-Rocha, F. Cádiz L. & D. Cádiz L. coll., 8 ma, 1 fe (MZSP 36799); Same, 2 ma, 1 fe (MZSP 36803); Same, Puesco, Parque Nacional Villarica (-39.565000, -71.513889), 22.I.2010, R. Pinto-da-Rocha, F. Cádiz L. & D. Cádiz L. coll., 9 ma, 6 fe (MZSP 36802); Región de Los Lagos, *Provincia de Osorno*, Pucatrihue, 23.III.1963, Tha & Lep coll., (AMNH); Same, Parque Nacional Puyehue, 12–22.II.1979, L.E. Peña coll., 2 ma, 4 fe (AMNH); Same, 5–12.II.1978, collector unknown, 1 ma (AMNH); Same (-40.666389, -72.171944), 15.I.2003, S.E. Lew & K.W. Will coll., 1 ma, 1 fe (CAS 9017709); Same, 31.I.1985, N.I. Platinick & O.F. Francke coll., 2 ma, 1 fe, 1 juv (AMNH); Same, Puyehue, 5–12.II.1978, collector unknown, 1 ma (AMNH); Same (-40.73715 -72.31062), 16.XI.2014, G. Giribet, G. Hormiga & A. Pérez-González coll., 2 ma, 2 fe (MCZ 138128); Same, Puyehue, Parque Nacional Puyehue (-40.665583, -72.175694), 30–31.XII.2016, A. Anker & P.H. Martins coll., 1 ma (UFMG 22633); Same, La Picada, 15–20.I.1980, L.E. Peña coll., 1 ma (AMNH); Same, Entre Lagos, 14–17.II.1978, collector unknown, 1 ma (AMNH); Same, 14–17.II.1978, collector unknown, 1 ma (AMNH); Same, *Provincia de Chiloé*, Ilha Chiloé, Chepu, 2.II.1985, N.I. Platnick & O.F. Francke coll., 1 ma (AMNH); Same, 20.II.2000, T. Cekalovic coll., 2 ma (AMNH); Same, Chepu, 14.II.2002, T. Cekalovic coll., 1 ma (AMNH); Same, Parque Nacional Chiloé (-42.574417, -74.077350), 3.XII.2009, H. Wood, L. Almeida & C. Griswold coll., 1 ma (CAS 9036299); Same, Termas de Puyehue (-40.7 -72.3; 250 masl), 12.III.1965, H. Levi coll., 4 ma (MCZ 38289); Pucatrihue (-40.54389 -73.71778; near the coast of Osorno), 3–21.III.1967, H. Levi coll., 6 ma, 2 fe (MCZ 38288); Same, XII.1967, L. Peña coll., 3 ma, 13 fe (MCZ 34212); Same, 2 ma (MCZ 38280); Same, 1 ma, 2 juvs (MCZ 38268); Same, 1 ma, 3 fe (MCZ 38282); Same, 1 ma, 2 fe (MCZ 38284); Same, 2 ma (MCZ 31244); Same, 1 ma (MCZ 31242); Same, 2 ma (MCZ 31245); Same, Parque Nacional Puyehue (-40.659722, -72.174167), 21.I.2010, R. Pinto-da-Rocha, F. Cádiz L. & D. Cádiz L. coll., 6 ma, 8 fe, 3 juv (MZSP 36800); Same (-40.659722, -72.174167), 20.I.2010, R. Pinto-da-Rocha, F. Cádiz L. & D. Cádiz L. coll., 1 ma, 1 fe (MZSP 36841); Same, *Provincia de Llanquihue*, Chamiza (-41.467 -72.85), 25.II.1976, T. Cekalovic coll., 4 ma, 6 fe (MCZ 31261); Same, Puerto Montt, Parque Nacional Alerce Andino (-41.593611, -72.593889), 15.XII.2010, F. Marques, F. Cadiz & F. Carbayo coll., 1 ma (MZSP 36842); Same (-41.59351, -72.59359), 15.XI.2014, G. Giribet, G. Hormiga & A. Pérez-González coll., 1 ma (MCZ 138113); Same, Monumento Nacional Lahuen Nadi (-37.810556, -73.057500), 14.XII.2010, F. Marques, F. Cadiz & F. Carbayo coll., 1 ma (MZSP 36837); Same, Los Muermos (-41.329167, -73.825278), 16.XII.2010, F. Marques, F. Cadiz & F. Carbayo coll., 1 ma, 2 fe (MZSP 36801); Región de Los Rios, *Provincia Valdivia*, Parque Oncol (-39.700250, -73.000000), 10.I.2007, E. Arias coll., 1 ma (CAS 9026268); Parque Nacional Oncol (-39.712333, -73.307278), 01.I.2017, A. Anker & P.H. Martins coll., 1 fe (UFMG 22634); Same, 1 fe (UFMG 22635); Same, 1 ma, 1 fe (UFMG 22636); Same, 1 ma (UFMG 22638); Same, 4 ma, 1 fe (UFMG 22639); Same, Rio Bueno, II.1945, R. Vaz-Ferreira coll., (MNHN 703; holotype of *S. exceptionalis*); Same, Neltume, I.1978, collector unknown, 1 ma, 1 fe (AMNH); Same, Parque Oncol, 7.II.2004, T. Cekalovic coll., 2 ma, 1 juv (AMNH); Same, 15.II.2004, T. Cekalovic coll., 2 ma, 2 fe (AMNH); Same, 7.II.2004, T. Cekalovic coll., 2 ma (AMNH); Same, 12.I.2006, Elizabeth Arias coll., 1 ma (AMNH); Same, 12.I.2006, Elizabeth Arias coll., 3 ma, 1 fe (AMNH); Same, Curiñanco, 15.II.2004, T. Cekalovic coll., 2 ma, 2 fe (AMNH); Same, Puerto Fuy, 16–20.II.1978, collector unknown, 1 ma (AMNH); Same (-39.81389 -73.24583), 15–20.XI.1978, E. Krahmer coll., 1 ma (MCZ); Same, Corral (-39.867 -73.433), 30.XII.1905, R. Thaxter coll., 1 ma (MCZ 38279); Same, date or collector unknown, 1 ma, 2 fe (MCZ 38286).

##### Diagnosis.

*Sadocus polyacanthus* resembles *S. dilatatus* by the posterior apophysis on the lateral margin of dorsal scutum, and femur IV almost straight. It is distinguished from the latter by the lack of a long, single retro-central apophysis (present in *S. dilatatus*) on femur IV, and by the presence of prolateral and retro-lateral rows of similar sized tubercles on femur IV and coxa IV being closer to the body (instead of spread to the sides as *S. dilatatus*).

##### Redescription.

**Male** (CAS 9055047). ***Measurements*.** Dorsal scutum maximum length 8.0; dorsal scutum maximum width 10.0; prosoma maximum length 3.3; prosoma maximum width 4.5; leg femora: I 4.9; II 9.6; III 8.2; IV 8.5. ***Dorsum*** (Fig. 7A). Dorsal scutum type gamma pyriform. Anterior margin of dorsal scutum with 15 granules, few closer to ocularium on carapace, lateral margin of carapace with a row of granules. Three scutal areas; scutal areas I–III with four, five, and eight granules, respectively. Scutal area III with one pair of spines. Lateral margin of dorsal scutum with row of granules increasing in size from the ozopore area to scutal area III, ending in a large spiniform tubercle. Posterior margin of dorsal scutum with a row of ten tubercles. ***Chelicerae*.** Segment I with a pair of retro-apical setae; fixed finger with three teeth, movable finger with four teeth. ***Pedipalps*.** Coxa with one central ventral spine. Trochanter with one ventro-apical tubercle. Femur with one ventro-basal seta and three ventro-basal granules. Patella covered with setae. Tibial setation: prolateral IiiIi/IiiiIi; retro-lateral: iIiIi. Tarsal setation: prolateral IiIii; retro-lateral IiIi. ***Legs*** (Fig. 7B–E). Trochanters I–IV granulate; trochanter II with one retro-lateral tubercle; trochanter III with one retro-apical spine; trochanter IV with one dorso-central tubercle, one retro-central tubercle, one retro-apical tubercle, one retro-dorsal apical spine; prodorsal apical apophysis with a rounded apex extending towards the dorsal region. Femur III ventral face with two rows of granules increasing in size apically. Femur IV with retro-dorsal row of spines decreasing in size towards the center; retro-lateral row of sparse spines increasing in size apically; ventro-apical face with a prolateral spine and retro-lateral granule. Patella IV with retro-lateral row of spines, the apical one curved, pointing towards tibia IV. Tibia IV with two retro-ventral apical spines. Tarsal counts: 6, 11, 7, 8. ***Penis*** (Fig. 11E, F). Ventral plate of penis with attenuated cleft on anterior margin; three pairs of MS A, two pairs of MS B, four or five pairs of MS C, two or three pairs of MS D.

***Coloration*.** Immersed in ethanol: carapace, trochanters I–IV and femur IV dark brown. Legs with a gradient from brown to caramel. Live specimens (Fig. 10C–F): carapace, patellae, and tibiae I–IV black, except the areas of dorsal scutum that can vary (yellow, orange or red). Coxa and trochanters black. Femora I–IV varying between black and orange. Posterior margin of dorsal scutum to free tergite III arthrodial membranes green.

***Variations*** (n = 58). Even in ethanol there is a great variation of coloration, which ranges from orange to caramel and yellow. The number of granules on the anterior margin of dorsal scutum varying from few sparsely distributed to completely covered. Measurements. Dorsal scutum maximum length 5.6–9.8; dorsal scutum maximum width 6.0–11.9; prosoma maximum length 2.2–4.0; prosoma maximum width 3.0–5.4; leg femora: I 3.5–6.9; II 6.4–13.0; III 5.3–11.4; IV 6.0–11.5.

**Female** (AMNH – 11- Chile, Region de Los Lagos, Oncol Parque, 12.I.2006, Luma Arias et al. coll., Berkeley). ***Measurements*.** Dorsal scutum maximum length 9.3; dorsal scutum maximum width 9.4; prosoma maximum length 3.6; prosoma maximum width 5.0; leg femora: I 6.0; II 11.5; III 9.5; IV 11.5. ***Dorsum*** (Fig. 8I, J). Scutal areas I and II with three and five granules, respectively. ***Legs*.** Coxa IV granulate, with short prodorsal apical spiniform apophysis, not visible in ventral view. Trochanter IV with one retro-lateral spiniform tubercle. Tarsal counts: 6, 11, 7, 8. ***Ovipositor*** (Fig. 12J–L). Two main groups of lobes delimited by constriction, ovipositors peripheral setae inserted into sockets that are a mixture of dorsal and ventral sockets, left lobe with four setae and right lobe with six; each main group of lobes divided by a fissure.

***Variations*** (n = 25). Color in dorsal scutum and femur varying between black and orange. Dorsal scutum with 10–30 granules. Measurements. Dorsal scutum maximum length 7.6–9.3; dorsal scutum maximum width 8.3–10.0; prosoma maximum length 3.0–3.6; prosoma maximum width 4.2–5.0; leg femora: I 4.5–6.0; II 7.0–13.0; III 7.0–11.4; IV 8.4–11.5.

##### Type locality.

Of *G. polyacanthus*: Chile. Provincias del Sur. Of *S. vitellinosulcatus*: either Australasia or South America. Of *G. platei*: Chile. Región de Los Lagos. *Provincia de Valdivia*. Corral. Of *A. exceptionalis* Chile. Región de Los Lagos. Osorno. Barra del Rio Bueno. Of *S. conspicillatus*: Chile. Región de Biobío. *Provincia de Concepción*. Concepción. Of *Sadocus guttatus*: Chile. Región de Biobío. *Provincia de Arauco*. Lebu.

##### Geographical distribution

**(Fig. 2).** Chile, *Región Metropolitana de Santiago*; Región de Biobío; Región de Araucanía; Región de Los Lagos; Ilha Chiloé; Región de Los Rios.

##### Taxonomic notes.

After examining the holotypes of *S. conspicillatus*, *S. guttatus*, and *S. exceptionalis*, we concluded that they were males within *S. polyacanthus* size variation. The apophyses size and shape on trochanter IV and the armature of femur IV (especially the retro-dorsal and retro-lateral row of spines size pattern) of all species are the same.

**Figure 9.**
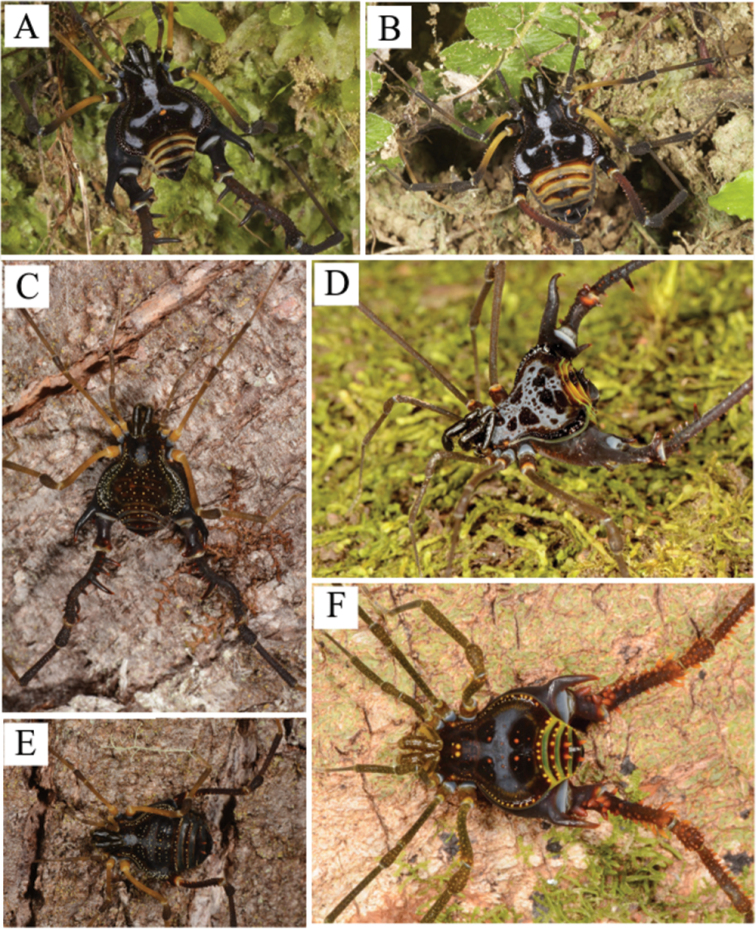
Photographs of living specimens of *Sadocus***A***S. asperatus* (ma) **B***S. asperatus* (fe) **C***S. asperatus* (ma) **D***S. dilatatus* (ma) **E***S. asperatus* (fe) **F***S. funestus* (ma). **A, B** taken by R. Pinto-da-Rocha, **C–F** taken by Pedro H. Martins.

**Figure 10.**
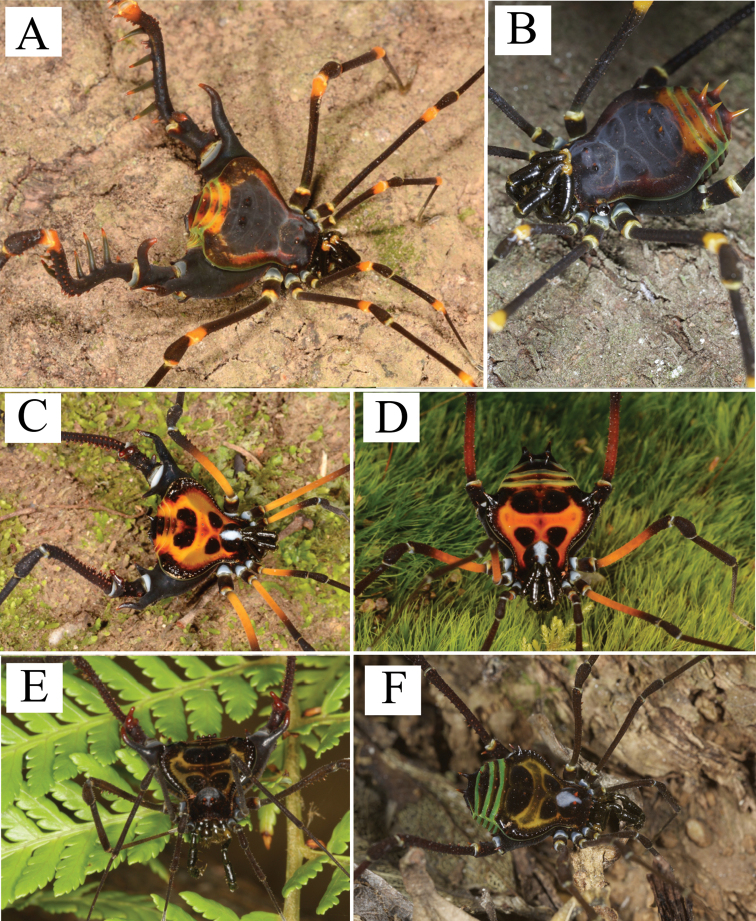
Photographs of living specimens of *Sadocus***A***S. ingens* (ma) **B***S. ingens* (fe) **C***S. polyacanthus* (ma) **D***S. polyacanthus* (fe) **E***S. polyacanthus* (ma) **F***S. polyacanthus* (fe). **B, E** taken by R. Pinto-da-Rocha; **A, C, D, F** taken by Pedro H. Martins.

### Species inquirendae

#### Sadocus allermayeri

Taxon classificationAnimaliaOpilionesGonyleptidae

(Mello-Leitão, 1945)

58C3DA4A-F326-595B-8CA8-23C16A1CEDA2

Carampangue allermayeri Mello-Leitão, 1945: 158 [desc]; Soares and Soares 1954: 241 [cat]; Cekalovic 1968: 7 [cat]; 1985: 16 [cat] (type material MNRJ, 1 ma & 1 fe syntypes, destroyed, not examined).
Sadocus allermayeri : Kury, 2003: 191 [cat]; Kury et al. 2020b [cat].

##### Type locality.

Chile. Región de Biobío. Concepción. Concepción.

##### Taxonomic notes.

The type material, belonging to MNRJ, was lost in the fire that destroyed most of the arachnid collection (Kury, pers. comm.; Kury et al. 2018). The original description is poor for modern standards and it has no illustrations. However, the description allows to be diagnosed by: the presence of scutal area IV on dorsal scutum, coxa IV with one prodorsal apical bifid apophysis; trochanter IV with one retro-basal apophysis and three proapical apophyses. *Sadocus asperatus*, *S. dilatatus*, and *S. ingens* have the scutal area IV in dorsal scutum and coxa IV with a bifid prodorsal apical apophysis; but none of them has three apical apophyses on trochanter IV. The only species with four scutal areas on the dorsal scutum and trochanter IV with three apical apophyses is *S. funestus*, but the prodorsal apical apophysis on coxa IV is uniramous. Assuming that the description is correct, it implies that this is a valid species that we have not yet located among the material gathered for this revision of *Sadocus*.

**Figure 11.**
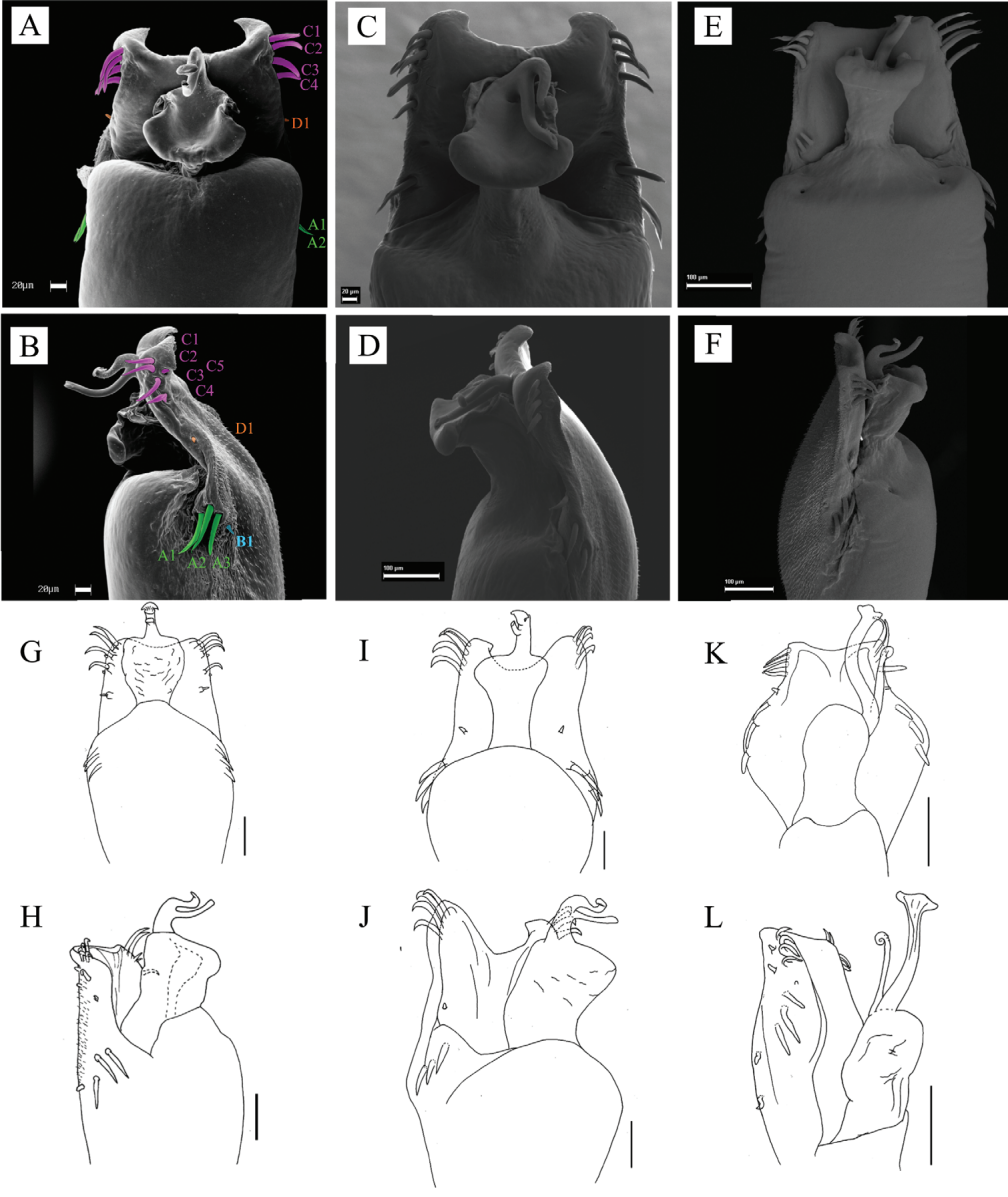
Distal part of penis *Sadocus* and *Discocyrtus*, dorsal and ventral views, respectively **A, B***S. asperatus*, macrosetae colorized and numbered **C, D***S. ingens***E, F***S. polyacanthus***G, H***S. dilatatus***I, J***S. funestus***K, L***D. catharinensis*. Scale bar of drawings: 0.1mm.

**Figure 12.**
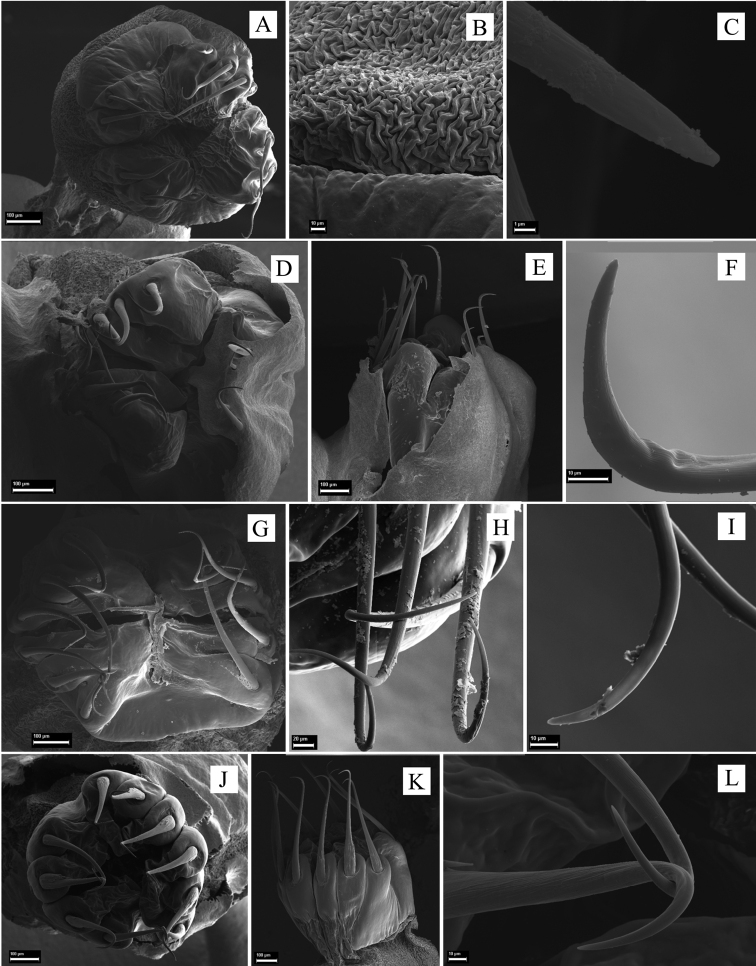
Distal part of ovipositor of *Sadocus***A–C***S. asperatus***A** upper view **B** villi **C** setae **D–F***S. funestus***D** upper view **E** lateral view **F** seta **G–I***S. ingens***G** upper view **H, I** setae **J–L***S. polyacanthus***J** upper view **K** lateral view **L** setae.

#### Sadocus nigronotatus

Taxon classificationAnimaliaOpilionesGonyleptidae

(Mello-Leitão, 1943)

8010DA6A-3D31-57FD-90EC-2B8521F71AE1

Figure 8K

Carampangue nigronotatum Mello-Leitão, 1943: 8, fig 7 [desc]; Soares and Soares 1954: 242 [cat]; Cekalovic 1968: 7 [cat]: 1985: 17 [cat] (type material MNRJ, 1 fe holotype lost, not examined).
Sadocus nigronotatus : Kury, 2003, 191 [cat]; Kury et al. 2020b [cat].

##### Type locality.

Chile. *Provincia de Llanquihue*. Región de Los Lagos. Maullín.

##### Taxonomic notes.

The type material, belonging to the MNRJ, is lost (for the same reason as *S. allermayeri*). The original description is poor for modern standards, and both description and figure are based on a female. The females of the different *Sadocus* species are very similar and difficult to identify unequivocally. According to the original description, *S. nigronotatus* resembles *S. polyacanthus* in the presence of a spiniform apophysis on the lateral margin of the dorsal scutum, but can be distinguished by the presence of scutal area IV.

### Species removed from *Sadocus*

#### Discocyrtus catharinensis

Taxon classificationAnimaliaOpilionesGonyleptidae

(Mello-Leitão, 1923)

ABA35B77-81CE-5BC4-9A34-D4550C96221E

Figure 11K, L

Sadocus catharinensis Mello-Leitão, 1923: 152 [desc]; B. Soares 1944a: 166 [syst]; 1944b: 222 [cit]; 1945: 364 [cat]; Soares and Soares 1949: 211 [cat]; H. Soares 1966: 90 [cat] (Types MNRJ 1510, syntypes examined by detailed photographs).
Parasadocus catharinensis : Mello-Leitão, 1927: 8 [syst]; Roewer 1930: 425 [rdesc]; Mello-Leitão 1932: 329 [rdesc].
Discocyrtus catharinensis : Soares & Soares, 1987: 458, figs 3–6 [syst, rdesc].
Sadocus aquifugus Mello-Leitão, 1931a: 136, fig 8 [desc]; 1935: 106 [cit] (Types MNRJ 11390 syntypes, male genitalia examined by photographs).
Gonyleptes pugilator Mello-Leitão, 1932: 303, fig 163 [desc]; B. Soares 1944b: 222 [cit]; Soares and Soares 1949: 180 [cat] (Type MNRJ – lost).
Gonyleptes acanthopus Mello-Leitão, 1945: 156 [cit] [nec Quoy and Gaimard 1824] – misidentification: Soares and Soares 1987].
Lycomedicus brasiliensis Soares & Soares, 1949: 52, figs 6–8 [desc]; 1954: 271 [cat] (type material – old collection CGPC, 1 ma, 1 fe – MZSP 36165, holotype, MZSP 1029 paratype, examined). syn. nov.
Sadocus brasiliensis : Kury, 2003: 191 [cat]; Kury et al. 2020b [cat].

##### Material examined.

Brazil, Paraná, *Piraquara*, Banhado, IV.1946, Goffergé coll., 1 ma holotype, 1 fe paratype (MZSP 36165); same, 1 ma paratype (MZSP 1029); Santa Catarina, *Joinville*, III.1947, Goffergé coll., 1 ma (MZSP 36419).

##### Type locality.

*S. catharinensis* and *S. aquifugus*: BRAZIL. Santa Catarina. Joinville. Of *G. pugilator*: BRAZIL. Santa Catarina. Of *Lycomedicus brasiliensis*: BRAZIL. Paraná. Piraquara: Banhado.

##### Taxonomic notes.

We examined the type material of *Sadocus brasiliensis* and its external and penial morphology did not match that of other Chilean species of the genus. Based on its type locality, we examined other Brazilian Pachylinae genera and found striking similarities between *S. brasiliensis* and *D. catharinensis*. We examined detailed pictures of the type material kindly shared by Rafael N. Carvalho and additional material from the MZSP collection. Those species are the same, and we propose that *S. brasiliensis* is the junior synonym of *D. catharinensis*. Many *Discocyrtus* spp. have been transferred to revalidated genera or newly created subfamilies, such as Roeweriinae (Carvalho and Kury 2018) or Neopachylinae (Carvalho and Kury 2021). Indeed, the penial features of *D. catharinensis* (Fig. 15K, L) resembles those of that subfamily, which is corroborated in the present analysis: *D. catharinensis* is the sister species of *R. bittencourti*. Based on male genitalia and leg IV (see Carvalho and Kury 2018), *D. catharinensis* seems to belong to *Discocyrtanus* Roewer, 1929. However, because there is an ongoing study revising *Discocyrtus* conducted by Rafael N. Carvalho (MNRJ) and taking into account that it will have serious taxonomic consequences, we opted to propose only the synonymy of *S. brasiliensis* with *D. catharinensis*.

#### Gonyleptes horridus

Taxon classificationAnimaliaOpilionesGonyleptidae

Kirby, 1819

DC8ABACC-23DF-5369-85E3-98D172DF8F75

Gonyleptes horridus Kirby, 1819: 452, pl. 22, fig 16 [desc]; Kury 2003: 128 [cat]; Hara et al. 2012: 38–39 [syst]; Pinto-da-Rocha et al. 2012: 26, 41–45 [syst]; Pinto-da-Rocha et al. 2014: 4, 17 [syst]; Hara 2016: 106–109 [syst]. (Type holotype NHM 1863.41, ma examined by detailed photographs).
Lycomedes calcar Roewer, 1913: 132, fig 59 [desc]; Acosta 1996: 218 [cit] (Type holotype SMF RI, 782, ma examined). syn. nov.
Lycomedicus calcar : Roewer, 1923: 444, fig 558 [rdesc]; Canals 1936: 69 [cat]; Soares and Soares 1954: 271 [cat]; Cekalovic 1968: 8 [cat], 1985: 18 [cat].
Sadocus calcar : Kury, 2003, 191 [cat]; Kury et al. 2020b [cat].

##### Taxonomic note.

The holotype of *Sadocus calcar* is in a very bad state of preservation; only part of the carapace, with the ozopores, and leg IV remain. The rest of the prosoma and all of the other legs are absent. Even in this condition, we noted that *S. calcar* lacks the large tubercles and spines on the free tergites, which are diagnostic of *Sadocus*. Furthermore, the armature of trochanter IV and the long, bifid, C-shaped dorso-basal apophysis on femur IV are strikingly similar to those of *Gonyleptes horridus*, a common species in the state of Rio de Janeiro. Therefore, we propose *S. calcar* as a junior synonym of *G. horridus*. This synonymy made us conclude that the provenance of *S. calcar* is mistaken, because *G. horridus* is endemic to the Brazilian Atlantic rainforest. It is widely known that Roewer, unfortunately, indicated wrong provenance of a few species, and this seems to be the case for this species.

#### Eubalta planiceps

Taxon classificationAnimaliaOpilionesGonyleptidae

(Gervais, 1842)

238C7919-F7AC-5E92-B3A1-9A4F53C0EC0A

##### Remarks.

*Sadocus planiceps* (originally *Gonyleptes planiceps* Gervais, 1842) has a convoluted taxonomic history, with many previously unknown synonyms detected, which will be published elsewhere (briefly commented in Pessoa-Silva et al. 2020). We excluded it from *Sadocus* because it lacks the synapomorphies of the genus. It also lacks the diagnostic characters of the genus, such as the typical ocularium shape and type of armature, dorsal scutum shape, just to name a few. Comparing with other species of Chilean Pachylinae, we detected striking similarities with *Eubalta meridionalis*. This synonymy did not go unnoticed by Kury et al. (2020a) in his catalogue, who also detected this in parallel with this revision. Finally, Kury et al. kindly invited us to publish this synonymy that resulted in a publication of that synonymy (Pessoa-Silva et al. 2020).

## Supplementary Material

XML Treatment for Sadocus

XML Treatment for Sadocus asperatus

XML Treatment for Sadocus dilatatus

XML Treatment for Sadocus funestus

XML Treatment for Sadocus ingens

XML Treatment for Sadocus polyacanthus

XML Treatment for Sadocus allermayeri

XML Treatment for Sadocus nigronotatus

XML Treatment for Discocyrtus catharinensis

XML Treatment for Gonyleptes horridus

XML Treatment for Eubalta planiceps

## References

[B1] Acosta LE (1996) Die Typus-Exemplare der von Carl-Friedrich Roewer beschrieben en Pachylinae (Arachnida: Opiliones: Gonyleptidae). Senckenbergiana biologica 76(1/2): 209–225.

[B2] Acosta LE (2002) Patrones zoogeográficos de los opiliones argentinos (Arachnida: Opiliones). Revista Ibérica de Aracnología 6: 69–84.

[B3] Acosta LE (2020) *Qorimayus*, a new genus of relictual, high-altitude harvestmen from western Argentina (Arachnida, Opiliones, Gonyleptidae) reveals trans-Andean phylogenetic links. Zootaxa 4722(2): 129–156. 10.11646/zootaxa.4722.2.2 32230630

[B4] Acosta LE Pérez-González A Tourinho AL (2007) Methods for taxonomic study. In: Pinto-da-Rocha R Machado G Giribet G (Eds) Harvestmen: The Biology of Opiliones.Harvard University Press, Cambridge, Massachusetts, 494–510.

[B5] Acosta LE Maury EA (1998) Opiliones. In: Morrone JJ Coscarón S (Eds) Biodiversidad de Artrópodos argentinos, Una perspectiva biotaxonómica.Ediciones Sur, La Plata, 569–580.

[B6] Amorim DS (1982) Classificação por sequenciação: uma proposta para a denominação dos ramos retardados. Revista Brasileira de Zoologia 1(1): 1–9. 10.1590/S0101-81751982000100001

[B7] Benavides LR Pinto-da-Rocha R Giribet G (2021) The phylogeny and evolution of the flashiest of the armored harvestmen (Arachnida: Opiliones). Systematic Biology, syaa080. 10.1093/sysbio/syaa080 33057723

[B8] Bremer K (1994) Branch support and tree stability. Cladistics 10: 295–304. 10.1111/j.1096-0031.1994.tb00179.x

[B9] Butler AG (1873) A monographic list of the species of the genus *Gonyleptes*, with descriptions of three remarkable new species. Annals and Magazine of Natural History (series 4) 11(62): 112–117. 10.1080/00222937308696775

[B10] Butler AG (1874) Descriptions of five new species of *Gonyleptes*. Zoological Journal of the Linnean Society 12: 151–155. 10.1111/j.1096-3642.1875.tb02579.x

[B11] Canals J (1934) Opiliones de la Argentina. Descripción de “*Diconospelta Gallardoi*”, n. gen., n. sp., y nómina de otros opiliones, nuevos para nuestro país. Estudios aracnológicos (V). 10.

[B12] Canals J (1936) Los Opiliones de Chile. Revista Chilena de Historia Natural 39: 68–71.

[B13] Carvalho RN Kury A (2018) Further dismemberment of *Discocyrtus* with description of a new Amazonian genus and a new subfamily of Gonyleptidae (Opiliones, Laniatores). European Journal of Taxonomy 393: 1–32. 10.5852/ejt.2018.393

[B14] Carvalho RN Kury AB (2021) A new subfamily of Gonyleptidae formed by false *Discocyrtus* Holmberg, 1878 from Brazil, with revalidation of *Pachylobos* Piza, 1940 and description of a new genus. Zoologischer Anzeiger 290: 79–112. 10.1016/j.jcz.2020.11.004

[B15] Cekalovic-K TN (1968) Conocimiento actual de los opiliones chilenos. Noticiario Mensual 12(138): 5–11.

[B16] Cekalovic-K TN (1976) Catalogo de los Arachnida: Scorpiones, Pseudoscorpiones, Opiliones, Acari, Araneae y Solifugae de la XII Region de Chile, Magallanes Incluyendo la Antartica Chilena (Chile). Gayana 37: 11–107.

[B17] Cekalovic-K TN (1985) Catálogo de los Opiliones de Chile (Arachnida). Boletin de la Sociedad de Biologia de Concepción 56: 7–29.

[B18] Cokendolpher JC (1993) Pathogens and parasites of Opiliones (Arthropoda: Arachnida). Journal of Arachnology 21(2): 120–146.

[B19] DaSilva MB Pinto-da-Rocha R (2010) Systematic review and cladistic analysis of the Hernandariinae (Opiliones: Gonyleptidae). Zoologia (Curitiba) 27(4): 577–642. 10.1590/S1984-46702010000400010

[B20] DaSilva MB Gnaspini P (2010) A systematic revision of Goniosomatinae (Arachnida: Opiliones: Gonyleptidae), with a cladistic analysis and biogeographical notes. Invertebrate Systematics 23(6): 530–624. 10.1071/IS09022

[B21] Gervais P (1842) Arachnides. Description et figures de quatre espèces nouvelles de Phalangiens. Magasin de Zoológie, Série 2, 4: 1–5.

[B22] Gervais P (1844) Acères Phrynéides, Scorpionides, Solpugides, Phalangides et Acarides; Dicères pizoïques, Aphaniptères et Thysanoures. In: Walckenaer CA (Org.) Histoire naturelle des Insectes Aptères 3: 94–131.

[B23] Gervais P (1847) Phalangides. In: Walckenaer CA, Gervais P (Eds) Histoire naturelle des Insectes Aptères 4: 344–345. [576–577.]

[B24] Gervais P (1849) Arácnidos. Orden IV Falangidos. In: Gay C (Org.) Historia fisica y politica de Chile, Zoologia IV: 18–2.

[B25] Goloboff PA Farris JS Nixon KC (2008) TNT, a free program for phylogenetic analysis. Cladistics 24: 774–786. 10.1111/j.1096-0031.2008.00217.x

[B26] Gray GR (1833) Arachnida. Animal Kingdom (Conversio Britannica operis illustris Cuvier) (Vol. 13). Whittaker, Treacher & Co., London, 540 pp.

[B27] Guérin-Méneville FÉ (1844) Iconographie du Règne-Animal de G. Cuvier: ou Représentation d’après nature de l’une des espèces les plus remarquables et souvent non encore figurées, de chaque genre d’animaux. 3. Texte explicatif (Vol. 3). Baillière, Paris, 930 pp.

[B28] Hara MR (2016) Cladistic analysis and description of three new species of the Chilean genus *Nanophareus* (Opiliones: Gonyleptidae: Pachylinae). Zootaxa 4105: 101–123. 10.11646/zootaxa.4105.2.1 27394767

[B29] Hara MR Pinto-Da-Rocha R (2010) Systematic review and cladistic analysis of the genus *Eusarcus* Perty 1833 (Arachnida, Opiliones, Gonyleptidae). Zootaxa 2698(1): 1–136. 10.11646/zootaxa.2698.1.1

[B30] Hara MR Pinto-da-Rocha R Kury AB (2012) Revision of *Nanophareus*, a mysterious harvestman genus from Chile, with descriptions of three new species (Opiliones: Laniatores: Gonyleptidae). Zootaxa 3579: 37–66. 10.11646/zootaxa.3579.1.2

[B31] Hogg HR (1913) 2. Some Falkland Island spiders. Proceedings of the Zoological Society of London 1913: 37–50. 10.1111/j.1096-3642.1913.tb01981.x

[B32] Holmberg EL (1878) Notas aracnologicas sobre los Solpugidos argentinos. El naturalista argentino 1(1): 69–74.

[B33] Kästner A (1937) Chelicerata. 7. Ordnung der Arachnida: Opiliones Sundeval = Weberknechte. In: Kukenthal W, Krumbach T (Eds) Handbuch der Zoologie 3(2): 300–393.

[B34] Kirby W (1819) A century of insects, including several new genera described from his Cabinet. Transactions of the Linnean Society of London 12(27): 375–453. 10.1111/j.1095-8339.1817.tb00239.x

[B35] Koch CL (1839) Uebersicht des Arachnidensystems 2. C. H. Zeh’sche Buchhandlung, Nürenberg, 38 pp.

[B36] Kury AB Carvalho RN (2016) Revalidation of the Brazilian genus *Discocyrtanus*, with description of two new species (Opiliones: Gonyleptidae: Pachylinae). Zootaxa 4111(2): 126–144. 10.11646/zootaxa.4111.2.2 27394903

[B37] Kury AB Giupponi APL Mendes AC (2018) Immolation of Museu Nacional, Rio de Janeiro – unforgettable ﬁre and irreplaceable loss. Journal of Arachnology 46: 556–558. 10.1636/JoA-S-18-094.1

[B38] Kury AB Villarreal MO (2015) The prickly blade mapped: establishing homologies and a chaetotaxy for macrosetae of penis ventral plate in Gonyleptoidea (Arachnida, Opiliones, Laniatores). Zoological Journal of the Linnean Society 174(1): 1–46. 10.1111/zoj.12225

[B39] Kury AB Medrano M (2016) Review of terminology for the outline of dorsal scutum in Laniatores (Arachnida, Opiliones). Zootaxa 4097(1): 130–134. 10.11646/zootaxa.4097.1.9 27394531

[B40] Kury AB Mendes AC Cardoso L Kury MS Granado Ade A (2020a) WCO-Lite: online world catalogue of harvestmen (Arachnida, Opiliones). Version 1.0 – Checklist of all valid nomina in Opiliones with authors and dates of publications up to 2018. Self published, Rio de Janeiro, 237 pp.

[B41] Kury AB Mendes AC Cardoso L Kury MS Granado Ade A Giribet G (2020b) World Catalogue of Opiliones. WCO-Lite version 1.2.1. https://wcolite.com/

[B42] Kury AB (1994) Early lineages of Gonyleptidae (ArachnidaOpilionesLaniatores). Tropical Zoology 7: 343–353. 10.1080/03946975.1994.10539264

[B43] Kury AB (2003) Annotated catalogue of the Laniatores of the New Word (Arachnida, Opiliones). Revista Ibérica de Aracnologia. Volumen especial monográfico 1: 5–337.

[B44] Loman JCC (1899) Die Opilioniden der Sammlung Plate. Zoologische Jahrbücher, Jena, Supplement, 5 (Fauna Chilensis. Zweiter Band), 1–14.

[B45] Maury EA (1991) Gonyleptidae (Opiliones) del bosque subantartico chileno-argentino I. El genero *Acanthoprocta* Loman, 1899. Boletin de la Sociedad de Biología de Concepción 62: 107–117.

[B46] Mello-Leitão CF (1923) OpilionesLaniatores do Brasil. Archivos do Museu Nacional 24: 107–197.

[B47] Mello-Leitão CF (1926) Notas sobre OpilionesLaniatores sul-americanos. Revista do Museu Paulista 14: 327–383.

[B48] Mello-Leitão CF (1927) Generos novos de Gony-leptideos. Boletim do Museu Nacional do Rio De Janeiro 3(2): 13–22.

[B49] Mello-Leitão CF (1931a) Opiliões novos ou criticos. Archivos do Museu Nacional 33: 115–145.

[B50] Mello-Leitão CF (1931b) Nota sobre arachnideos argentinos. III. Opiliões novos ou críticos. IV. Aranhas novas. Annaes da Academia brasileira de Sciencias 3(2): 83–97.

[B51] Mello-Leitão CF (1932) Opiliões do Brasil. Revista do Museu Paulista 17(2): 1–505.

[B52] Mello-Leitão CF (1935) Algumas notas sobre os Laniatores. Archivos do Museu Nacional 36(4): 87–116.

[B53] Mello-Leitão CF (1937) Cuatro géneros nuevos de Pachylinae. Revista Chilena de Historia natural 41: 149–156.

[B54] Mello-Leitão CF (1939) Les arachnides et la zoogeographie de l’Argentine. Physis 17: 601–630.

[B55] Mello-Leitão CF (1943) Aracnidos de Maullín. Revista Chilena de Historia natural 46: 1–8.

[B56] Mello-Leitão CF (1945) Considerações sobre o gênero *Eusarcus* Perty e descrição de quatro novos Laniatores. Anais Academia brasileira de Ciências 7(2): 149–162.

[B57] Mello-Leitão CF (1946) Nuevos aracnidos sudamericanos de las colecciones del Museo de Historia Natural de Montevideo. Comunicaccones Zoologicas del Museu de Historia natural de Montevideo 35(2): 1–11.

[B58] Mello-Leitão CF (1949) Famílias, subfamília, espécies gêneros novos de opiliões e notas de sinonimia. Boletim do Museu Nacional 94: 1–33.

[B59] Moritz M (1971) Die Typen der Arachniden-Sammlung der zoologischen Museums Berlin I. Opiliones. Mitteilungen aus dem Zoologischen Museum in Berlin 47(1): 189–214. 10.1002/mmnz.19710470116

[B60] Muñoz-Cuevas A (1973) Sur les caractères génériques de la famille des Gonyleptidae (Arachnida, Opiliones, Laniatores). Bulletin du Muséum National d’Histoire Naturelle 87(113): 225–234.

[B61] Nixon KC (1999) Winclada (beta) ver. 0.9. Published by the author, rearrangements are optimal for slight perturba. Ithaca. http://www.cladistics.com

[B62] Pérez-González A Cotoras DD Acosta LE (2020) Early detection of an invasive harvestman in an oceanic island? Remarkable findings of *Parabalta reedii* (Opiliones, Gonyleptidae) in the Juan Fernández archipelago, Chile. Studies on Neotropical Fauna and Environment, 8 pp. 10.1080/01650521.2020.1809611

[B63] Pérez-Schultheiss J Urra F Otárola A (2019) OpilionesLaniatores (Arachnida) de la Cordillera de Nahuelbuta: un desconocido hotspot de diversidad. Boletín Nahuelbuta Natural 4: 1–24.

[B64] Perty M (1833) Delectus animalium articulorum quae in itinere per Brasilia annni 1817–1820 peracta collegerunt J.B. Spix et de Martius, Monachii, 205 pp.

[B65] Pessoa-Silva M Hara M Pinto-da-Rocha R (2020) Chapter 12. Pachylinae at their southernmost extreme (Laniatores: Gonyleptidae). In: Kury AB Mendes AC Cardoso L Kury MS Granado AA (Eds) WCO-Lite: online world catalogue of harvestmen (Arachnida, Opiliones).Version 1.0 – Checklist of all valid nomina in Opiliones with authors and dates of publication up to 2018. Self published, Rio de Janeiro, 58–59.

[B66] Pinto-da-Rocha R Bragagnolo C (2010) Review of the Brazilian Atlantic Rainforest harvestman *Longiperna* (Opiliones: Gonyleptidae: Mitobatinae). Zoologia (Curitiba) 27(6): 993–1007. 10.1590/S1984-46702010000600023

[B67] Pinto-da-Rocha R (1997) Systematic review of the Family Stygnidae (Opiliones: Laniatores: Gonyleptoidea). Arquivos de Zoologia 33(4): 163–342. 10.11606/issn.2176-7793.v33i4p163-342

[B68] Pinto-Da-Rocha R (2002) Systematic review and cladistic analysis of the Caelopyginae (Opiliones, Gonyleptidae). Arquivos de Zoologia 36(4): 357–464.

[B69] Pinto-da-Rocha R Benedetti A Vasconcelos E Hara M (2012) New systematic assignments in Gonyleptoidea (Arachnida, Opiliones, Laniatores). ZooKeys 198: 25–68. 10.3897/zookeys.198.2337 PMC3368255 22707905

[B70] Pinto-da-Rocha R Bragagnolo C Marques FPL Junior MA (2014) Phylogeny of harvestmen family Gonyleptidae inferred from a multilocus approach (Arachnida: Opiliones). Cladistics 20(5): 519–539. 10.1111/cla.12065 34772271

[B71] QGIS (2019) QGIS Geographic Information System. Open Source Geospatial Foundation Project. http://qgis.org

[B72] Quoy JRC Gaimard JP (1824) Division 3° Zoologie. Section II. Des Arachnides et des Insectes. In: Freycinet L de (Ed.) Voyage autour du Monde entrepris par ordre du Roi exécuté sur les corvettes de S.M. l’Uranie et la Physicienne, pendant les années 1817, 1818, 1819 et 1820. Pillet Aîné, Paris, 542–558.

[B73] Ringuelet RA (1955a) Ubicación zoogeográfica de las Islas Malvinas. Revista del Museo de La Plata 6(48): 419–464.

[B74] Ringuelet RA (1955b) Vinculaciones faunisticas de la zona boscosa de Nahuel Huapi y el domino zoogeografico austral-Cordillerano. Notas del Museo de La Plata 18(160): 81–121.

[B75] Ringuelet RA (1957) Bioeografia de los aracnidos Argentinos del orden Opiliones. Contribuciones Cientificas Facultad Ciencias Exactas y Naturales Universidad de Buenos Aires, Serie Zoologia 1(1): 1–33.

[B76] Ringuelet RA (1959) Los aracnidos Argentinos del orden Opiliones. Revista del Museo Argentino de Ciencias Naturales “Bernardino Rivadavia.” Ciencias Zoologicas 5(2): 127–439.

[B77] Roewer CF (1913) Die Familie der Gonyleptiden der Opiliones-Laniatores. Archiv für Naturgeschichte. 79A(4–5): 1–473.

[B78] Roewer CF (1923) Die Weberknechte der Erde. Systematische Bearbeitung der bisher bekannten Opiliones. Gustav Fischer, Jena, 1116 pp.

[B79] Roewer CF (1925) Opilioniden aus Süd-Amerika. Bollettino dei Musei di Zoologia e di Anatomia Comparata della Reale Università di Torino 40(34): 1–34.

[B80] Roewer CF (1929) Weitere Weberknechte III. (3. Ergänzung der: Weberknechteder Erde”, 1923). Abhandlungen der Naturwissenschaftlichen Verein zu Bremen 27(2): 179–284.

[B81] Roewer CF (1930) Weitere Weberknechte IV. (4. Ergänzung der Weberknechte der Erde, 1923). Abhandlungen der Naturwissenschaftlichen Verein zu Bremen 27(3): 341–452.

[B82] Roewer CF (1938) Opiliones aus dem Naturhistorischen Reichsmuseum in Stockholm. Arkiv för zoologi, Series B. 30(10): 1–8. 10.5962/bhl.part.1498

[B83] Roewer CF (1943) Über Gonyleptiden. Weitere Webernechte (Arachn., Opil.) XI. Senckenbergiana 26(1–3): 12–68.

[B84] Roewer CF (1961) Opiliones aus Süd-Chile. Senckenbergiana Biologica 42(1/2): 99–105.

[B85] Simon E (1879) Essai d’une classification des Opiliones Mecostethi. Remarques synonymiques et descriptions d’espèces nouvelles. Première partie. Annales de la Société Entomologique de Belgique 22: 183–241.

[B86] Simon E (1884) Arachnides recueillis par la mission du Cap Horn en 1882–1883. Bulletin de la Société Zoologique de France, Paris 9: 117–144.

[B87] Soares HEM Soares BAM (1987) Opera Opiliologica Varia XVIII. (Opiliones, Cosmetidae e Gonyleptidae). Revista Brasileira de Entomologia 31(1): 1–11.

[B88] Soares HM Soares BA (1985) Opera Opiliologica Varia XXII. OpilionesGonyleptidae. Naturalia 10: 157–200.

[B89] Soares BAM (1944a) Notas sobre opiliões da coleção do Museu Nacional do Rio de Janeiro. Papéis avulsos do Departamento de Zoologia 6(15): 163–180.

[B90] Soares BAM (1944b) Notas sobre opiliões XIV. Papéis avulsos do Departamento de Zoologia 6(20): 221–224.

[B91] Soares BAM (1945) Opiliões da coleção do Museu Nacional do Rio de Janeiro. Arquivos de zoologia do Estado de São Paulo 4(9): 341–394.

[B92] Soares BAM Soares HEM (1954) Monografia dos gêneros de opiliões neotrópicos III. Arquivos de zoologia do Estado de São Paulo 8(9): 225–302.

[B93] Soares BAM Soares HEM (1949) Monografia dos gêneros de opiliões neotrópicos II. Arquivos de zoologia do Estado de São Paulo 7(2): 149–240.

[B94] Soares BAM (1945) Opiliões da coleção do Museu Nacional do Rio de Janeiro. Arquivos de zoologia do Estado de São Paulo 4(9): 341–394.

[B95] Soares HEM (1966) Opiliões da coleção Gofferjè (Opiliones: Gonyleptidae, Phalangodidae). Papéis Avulsos do Departamento de Zoologia 18(10): 77–102.

[B96] Soares HEM (1968) Contribuição ao estudo dos opiliões do Chile (Opiliones: Gonyleptidae, Triaenonychidae). Papéis avulsos de Zoologia 21(27): 259–272.

[B97] Sørensen WE (1884) OpilionesLaniatores (Gonyleptides W.S. Olim) Musei Hauniensis. Naturhistorisk Tiddskrift 14(3): 555–646.

[B98] Sørensen WE (1886) Opiliones. In: Koch L Keyserling E von (Eds) Die Arachniden Australiens nach der Natur beschrieben und abgebildet, vol.[Theil] 2 of two volumes & atlas, 1871–1889, fascicle [Lieferung] 33. Bauer & Raspe, Nürnberg, 53–86.

[B99] Sørensen WE (1902) Gonyleptiden (Opiliones, Laniatores). Ergebnisse der Hamburger Magalhaensischen Sammelreise 6(5): 1–36.

[B100] Strand E (1942) Miscellanea nomenclatoria zoologica et Paleontologica X. Folia zoologica et hydrobiologica 11(1): 386–402.

[B101] Sundevall CJ (1833) Conspectus Arachnidum. C.F. Berling, Londini Gothorum [Lund (Sweden)], 39 pp.

[B102] Townsend VR Bertram MS Milne MA (2015) Variation in ovipositor morphology among laniatorean harvestmen (Arachnida: Opiliones). Zoomorphology 134(3): 487–497. 10.1007/s00435-015-0269-4

